# Tim-3-targeted vaccines overcome tumor immunosuppression and reduce cDC1 dependence to elicit potent anti-tumor immunity

**DOI:** 10.1073/pnas.2518080123

**Published:** 2026-03-19

**Authors:** Chunmei Fu, Tianle Ma, Björn E. Clausen, Ira Mellman, Aimin Jiang

**Affiliations:** ^a^Center for Cutaneous Biology and Immunology, Department of Dermatology, Henry Ford Health, Detroit, MI 48202; ^b^Immunology Program, Henry Ford Cancer Institute, Henry Ford Health, Detroit, MI 48202; ^c^Department of Medicine, College of Human Medicine, Michigan State University, East Lansing, MI 48824; ^d^Department of Computer Science and Engineering, School of Engineering and Computer Science, Oakland University, Rochester, MI 48309; ^e^Institute for Molecular Medicine and Research Center for Immunotherapy, University Medical Center of the Johannes Gutenberg-University, Mainz 55131, Germany; ^f^Department of Biochemistry and Biophysics, School of Medicine, University of California, San Francisco, CA 94143; ^g^Parker Institute for Cancer Immunotherapy, San Francisco, CA 94129

**Keywords:** dendritic cells, type 1 conventional DCs, type 2 conventional DCs, Tim-3-targeted vaccines, anti-tumor CD8 T cell immunity

## Abstract

Conventional type 1 dendritic cells (cDC1s) are specialized for cross-presenting tumor antigens and determining the efficacy of immunotherapies, including immune checkpoint blockade and adoptive cell therapy. However, their rarity and tumor-induced dysfunction severely limit CD8 T cell priming and represent a central bottleneck to therapeutic efficacy. While strategies such as anti–DEC-205-mediated antigen delivery and Flt3L-driven DC expansion can enhance host DC function, their reliance on functional cDC1s remains a significant constraint. We developed Tim-3-targeted vaccines by conjugating tumor antigens or neoantigens to anti–Tim-3 antibodies. These vaccines delivered antigens to both cDC1s and cDC2s, and elicited robust, durable CD8 T cell responses. Remarkably, Tim-3-targeted vaccines endowed cDC2s with efficient cross-presentation capacity that matched that of cDC1s. In tumor-bearing mice or in CD11c-β-catenin^active^ mice, which model β-catenin-driven DC dysfunction, Tim-3-targeted vaccination restored cross-priming and counteracted tumor- and DC-mediated immunosuppression. In Batf3^−/−^ mice lacking cDC1s, anti-Tim-3-based vaccines still elicited significant CD8 T cell cross-priming and tumor control—albeit both were reduced compared to wild-type mice— demonstrating that cDC1s contribute to but are not essential for Tim-3-targeted vaccine–induced CD8 T cell priming and anti-tumor efficacy. Strikingly, a single dose of anti-Tim-3–neoantigen vaccination eradicated large established MC38 tumors in a CD8 T cell–dependent manner. Together, these data identify Tim-3-targeted vaccines as a next-generation cancer vaccine platform that broadens DC engagement, reduces reliance on cDC1s, and overcomes tumor- and DC-mediated immunosuppression, addressing key limitations of current DC-based cancer vaccines.

## Significance

The limited efficacy of DC vaccines and immune checkpoint blockade (ICB) partly reflects the scarcity and dysfunction of cDC1s in tumors. Tim-3-targeted vaccines deliver antigens to Tim-3^+^ antigen-presenting cells, including both cDC1s and cDC2s, and elicit robust, durable CD8 T cell responses. Tim-3-targeted vaccination enhances cross-priming in tumor-bearing and DC-impaired mice and counteracts tumor- and DC-mediated immunosuppression. Although cDC1 deficiency reduces CD8 T cell responses, Tim-3-targeted vaccines retain anti-tumor activity when cDC1s are genetically absent, demonstrating that cDC1s contribute to but are not strictly required for efficacy. A single dose of Tim-3–neoantigen vaccine eradicated large tumors in a CD8-dependent manner, offering a strategy to overcome immunotherapy barriers and expand treatment options for patients unresponsive to current therapies.

Dendritic cells (DCs) are crucial for the efficient cross-presentation of tumor antigens to activate tumor antigen-specific CD8 T cells—a process known as cross-priming (1, 2)—which is critical for effective tumor control (3–5). Among DC subsets, type 1 conventional DCs (cDC1s) are uniquely specialized for cross-presentation and play a central role in generating anti-tumor CD8 T cell responses and determining the efficacy of cancer immunotherapies, including immune checkpoint blockade (ICB) and adoptive cell therapy (ACT) (6–10). This specialized functionality of DCs makes DC-based vaccines one of the leading strategies for cancer immunotherapy (11, 12). However, current DC-based vaccines have been largely unsuccessful, and a major barrier is their reliance on host DCs—especially the rare and often impaired cDC1 subset—for antigen cross-presentation to induce optimal CD8 T cell immunity (13, 14). Tumors often target cDC1s to inhibit their infiltration into the tumor microenvironment (TME) or to render them dysfunctional and/or suppressive, thereby impairing T cell activation and limiting efficacy of immunotherapies including ICB and DC vaccines (7–10). Strategies like targeted antigen delivery (e.g., anti-DEC-205-antigen) or Flt3L-induced DC expansion can enhance efficacy (15–18), but their reliance on functional cDC1s—especially in the immunosuppressive TME--remains a significant barrier. Given the encouraging clinical outcomes of neoantigen-based DC vaccines (19), there is a pressing need to develop new vaccine strategies that could bypass cDC1 dependence and overcome tumor/DC-mediated immunosuppression, either as monotherapies or in combination with other therapies such as ICB.

Recent studies have identified Tim-3 (T cell immunoglobulin and mucin-domain containing-3), a checkpoint receptor originally described on IFN-γ-producing T cells (20), as being highly expressed on tumor-associated DCs, particularly cDC1s (21–23). Importantly, Tim-3 on cDC1s has been shown to mediate the effects of anti-Tim-3 antibody treatment in improving anti-tumor efficacy of chemotherapy and DC vaccines (21, 24). In line with these studies, a recent report further demonstrates that Tim-3 on DCs instead of T cells (both CD4 and CD8 T cells) is essential for mediating the beneficial effects on anti-tumor immunity by Tim-3 blockade, highlighting a DC-intrinsic mechanism (25). Consistent with the notion that anti–Tim-3 enhances DC function, Tim-3 blockade in CD11c-β-catenin^active^ mice--which model tumor-induced β-catenin-mediated DC dysfunction—restores cross-priming induced by cDC1 (DEC-205)-targeted vaccination and, when combined with DEC-205-targeted vaccines, further improves anti-tumor efficacy (26). Notably, Tim-3 is also upregulated in tumor-associated cDC2s across several tumor models (22, 27), suggesting broader induction across DC subsets. In support of this, RNA-sequencing of DCs with active β-catenin (from CD11c-β-catenin^active^ mice)—which mimic tumor-induced β-catenin activation in DCs (28)—reveals that β-catenin upregulates Tim-3 across multiple DC subsets, including cDC2 populations, in addition to cDC1s (29). DC-targeted vaccines using anti–DEC-205–antigen conjugates enhance antigen cross-presentation by more than 1,000-fold compared to immunization with soluble antigens (15, 16, 30), but their efficacy still depends on functional cDC1s. Given that Tim-3 is broadly expressed across DC subsets, we reasoned that targeting Tim-3 with anti-Tim-3 antibodies engineered to carry tumor antigens could simultaneously block Tim-3 signaling on DCs—thereby enhancing DC function (21, 24, 25), and deliver antigens to multiple Tim-3^+^ APC populations including cDC1s and cDC2s. This dual-action strategy should reduce reliance on cDC1s and potentially sustain cross-priming and CD8 T cell immunity even when cDC1s are scarce or dysfunctional.

In this study, we developed a Tim-3-targeted vaccination strategy by conjugating diverse antigens to anti–Tim-3 antibodies and evaluated their efficacy—including ovalbumin (OVA peptide and protein), the melanoma-associated antigen gp100, the MC38 neoantigen Adpgk, and the Lewis Lung Carcinoma (LLC) neoantigen mRiok1. Tim-3-targeted vaccines elicited strong CD8 T cell responses and restored cross-priming in settings of both β-catenin-mediated DC dysfunction and established tumor-mediated immunosuppression. Notably, Tim-3-targeted vaccination still achieved substantial CD8 T cell cross-priming and anti-tumor efficacy in Batf3^−/−^ mice lacking cDC1s, albeit at reduced levels than in wild type (WT) controls, indicating that alternative Tim-3^+^ APCs can compensate for cDC1 deficiency. Vaccination with anti–Tim-3hgp100 outperformed the benchmark cDC1-targeted anti–DEC-205hgp100 vaccine combined with anti-Tim-3 ICB. Likewise, anti-Tim-3-based neoantigen vaccine against LLC (anti-Tim-3mRiok1) elicited strong anti-tumor efficacy in both WT and Batf3^−/−^ mice, contrasting with prior reports that mRiok1 peptide vaccination failed to control LLC even in combination with anti–CTLA-4 or anti–PD-1 therapy (31). Strikingly, a single dose of anti–Tim-3Adpgk eradicated large established MC38 tumors, achieving efficacy superior to conventional DC-targeting vaccination and ICB regimens in this model (22, 32, 33). Collectively, these data establish Tim-3-targeted vaccines as a versatile platform that broadens APC participation—including cDC2s—to drive potent CD8 T cell–mediated anti-tumor immunity and tumor control, mitigating reliance on cDC1s by retaining efficacy even when cDC1s are absent, and overcoming tumor- and DC-imposed barriers to anti-tumor immunity.

## Results

### Tim-3-Targeted Vaccines with Model Antigen OVA Induce Robust Cross-Priming and Overcome DC (β-Catenin)-Mediated Immunosuppression.

As Tim-3 on DCs has been shown to play an essential role for anti-tumor efficacy of Tim-3 blockade (25), and DC-targeted antigen delivery is known to enhance cross-presentation by the targeted DCs (16, 30), we thus investigated whether targeting antigens to Tim-3-expressing antigen-presenting cells (APCs) using an anti–Tim-3 antibody would efficiently cross-prime antigen-specific CD8 T cell responses by employing the cross-presentation capacity of cDC1s and other Tim-3-expressing DC subsets.

We conjugated anti-Tim-3 or isotype control monoclonal antibodies to the model antigen chicken ovalbumin (OVA) for MHCI, using a long peptide encompassing the MHCI (H-2 K^b^) epitope SIINFEKL (TRE*SIINFEKL*EKCK-Biotin, referred to as anti-Tim-3OTIp). Western blot analysis confirmed that the conjugated antibody carried the biotin-tagged long peptide (*SI Appendix*, Fig. S1). Next, C57BL/6 WT mice were adoptively transferred with naive OVA-specific Thy1.1^+^ CD8 T (OTI) cells, and immunized with either anti-Tim-3OTIp or isotypeOTIp with poly I:C and CpG as adjuvants. Immunization with anti-Tim-3OTIp led to robust proliferation of OTI cells, resulting in significantly higher frequencies of Thy1.1^+^ OTI out of total CD8 T cells, as well as higher percentages of Thy1.1^+^ OTI that had undergone 6 or more cycles of proliferation, compared to mice immunized with isotypeOTIp (*SI Appendix*, Fig. S2 *A* and *B*). As expected, primed OTI cells differentiated into effector cells following immunization of anti-Tim-3OTIp, with the majority of primed OTI cells becoming IFN-γ-producing effectors (*SI Appendix*, Fig. S2*C*).

**Fig. 1.**
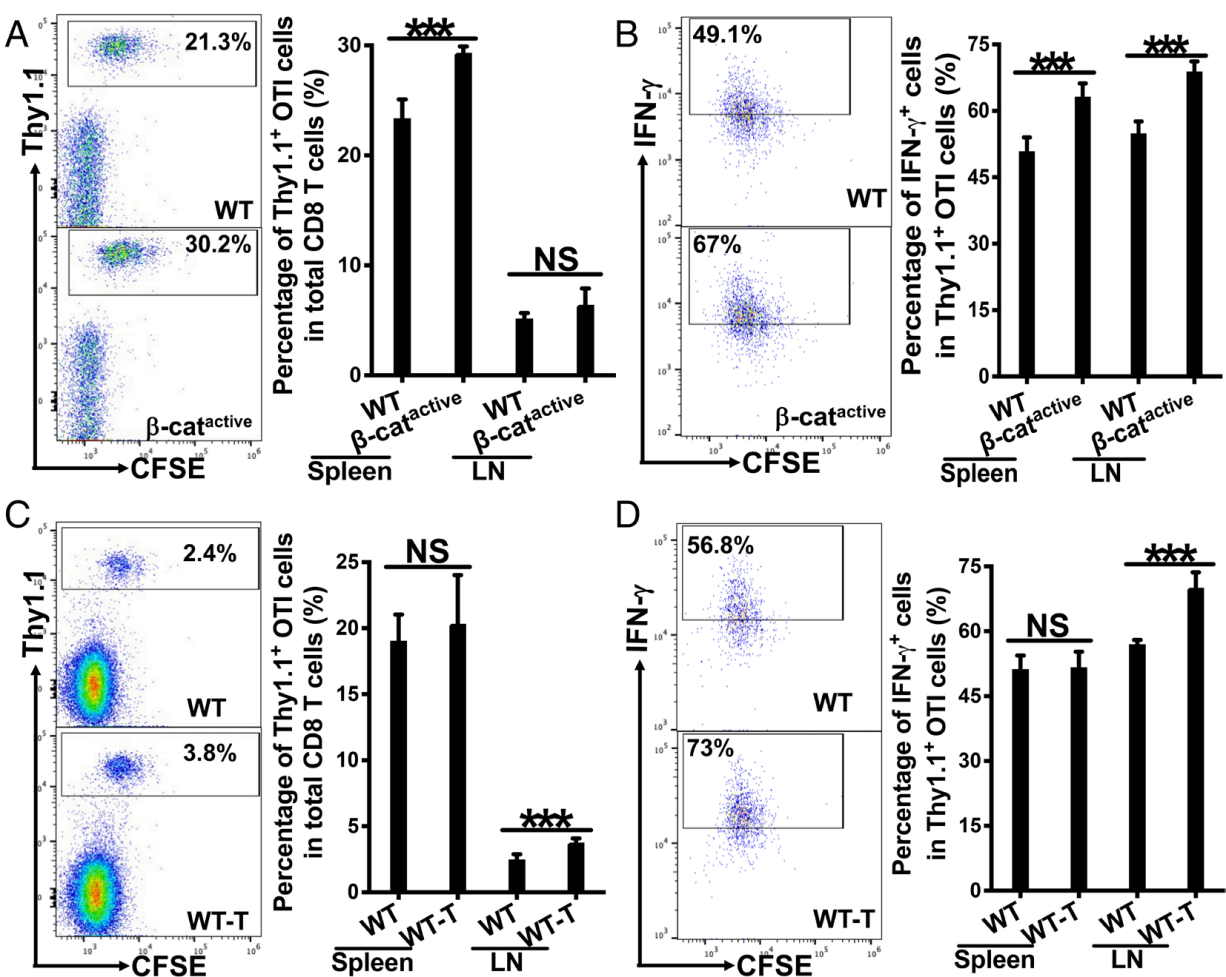
Anti-Tim-3OTIp vaccine-induced cross-priming is resistant to β-catenin- and tumor-mediated immunosuppression. (*A* and *B*) Vaccination with anti-Tim-3OTIp induced comparable or higher cross-priming in CD11c-β-catenin^active^ mice than WT mice. WT and CD11c-β-catenin^active^ mice (n = 4 to 5) were immunized with anti-Tim-3OTIp with CpG and Poly I:C as adjuvants. Cross-priming of adoptively transferred Thy1.1^+^ OVA-specific CD8 T (OTI) cells was examined at day 3 after immunization. (*A*) The percentages of Thy1.1^+^ OTI cells in total CD8 T cells are shown with representative dot plots from flow cytometry of splenocytes (*Left*) and bar graph (*Right*), (*B*) the percentages of IFN-γ^+^ effectors out of total Thy1.1^+^ OTI cells are shown with representative histogram from flow cytometry of splenocytes (*Left*) and bar graph (*Right*). (*C* and *D*) Vaccination with anti-Tim-3OTIp induced comparable or stronger cross-priming in B16OVA-bearing mice compared to tumor-naive WT mice. Tumor-naive and B16OVA-bearing WT mice (n = 4 to 5) were immunized with anti-Tim-3OTIp as above. The percentages of Thy1.1^+^ OTI cells in total CD8 T cells (*C*) with representative dot plots from LN flow cytometry (*Left*) and bar graph (*Right*), and the percentages of IFN-γ^+^ effectors out of total Thy1.1^+^ OTI cells (*D*) are shown with representative dot plots from LN flow cytometry (*Left*) and bar graph (*Right*). Student’s *t* tests, ^***^P < 0.001, and NS > 0.05. Data are representative of at least two experiments.

Previously, we demonstrated that activation of β-catenin signaling in DCs (using CD11c-β-catenin^active^ mice) impaired cross-priming upon cDC1-targeted vaccination with anti–DEC-205–antigen conjugates (26, 28). More recently, we have reported that β-catenin activation upregulates Tim-3 expression across multiple DC subsets, including multiple cDC2 populations, in addition to cDC1s (29). Based on these findings, we next investigated whether targeting antigens to these Tim-3^+^ DCs using anti-Tim-3OTIp could overcome β-catenin-mediated immunosuppression. WT and CD11c-β-catenin^active^ mice were adoptively transferred with Thy1.1^+^ OTI cells, and immunized with anti-Tim-3OTIp plus adjuvants as above. Interestingly, the percentages of Thy1.1^+^ OTI out of total CD8 T cells in CD11c-β-catenin^active^ mice were higher than the percentages in WT mice (Fig. 1*A*). Notably, OTI cells primed in CD11c-β-catenin^active^ mice exhibited significantly higher frequencies of differentiated IFN-γ^+^ effectors compared to WT mice (Fig. 1*B*). This contrasts with impaired cross-priming (i.e., substantially lower percentages of OTI cells and IFN-γ^+^ effectors) observed in CD11c-β-catenin^active^ mice compared to WT mice upon cDC1-targeted vaccination with anti-DEC-205OVA (28). Moreover, CD11c-β-catenin^active^ mice exhibited significantly higher levels of IFN-γ^+^IL-2^+^ polyfunctional OTI effectors, which are critical for CD8 T cell memory responses (34, 35), as well as IFN-γ^+^TNFα^+^ polyfunctional OTI effectors, compared to WT mice (*SI Appendix*, Fig. S3 *A* and *B*).

Collectively, these findings demonstrate that Tim-3-targeted vaccines overcome β-catenin (DC)-mediated immunosuppression, supporting effective cross-priming and effector differentiation of CD8 T cells.

### Tim-3-Targeted OVA Vaccines Overcome Tumor-Mediated Immunosuppression and Induce Robust Cross-Priming of Antigen-Specific CD8 T Cells.

Our previous work demonstrated that B16OVA-bearing mice exhibit elevated β-catenin in DCs and impaired CD8 T cell priming when vaccinated with cDC1-targeted anti–DEC-205OVA, resulting in substantially reduced frequencies of OTI cells and IFN-γ^+^ effector compared to tumor-naive WT mice (28). Tim-3 is highly expressed by tumor-associated cDC1s, and recent studies also report elevated Tim-3 expression on cDC2s in multiple tumor models (21, 22, 27), suggesting that Tim-3 upregulation is not restricted to cDC1s.

To assess whether Tim-3 upregulation occurs systemically beyond the TME, we analyzed Tim-3 expression on splenic cDC subsets in B16F10-bearing WT mice (*SI Appendix*, Fig. S4*A*). The spleen provides a well-defined and accessible source of DCs for phenotypic analysis, enabling us to assess systemic changes in Tim-3 expression. As expected, cDC1s expressed higher levels of Tim-3 than cDC2s in tumor-naive mice (*SI Appendix*, Fig. S4*B*). Importantly, both cDC1s and cDC2s exhibited significant Tim-3 upregulation in tumor-bearing mice relative to tumor-naive mice (*SI Appendix*, Fig. S4*B*).

These findings prompted us to test whether Tim-3-targeted vaccines could resist tumor-mediated immunosuppression. B16OVA-bearing and tumor-naive WT mice were immunized with anti-Tim-3OTIp plus adjuvants (Poly I:C and CpG) following adoptive transfer of Thy1.1^+^ OTI cells. Strikingly, B16OVA-bearing mice showed comparable (spleen) or even higher (lymph node) frequencies of Thy1.1^+^ OTI cells out of total CD8 T cells compared to tumor-naive WT mice (Fig. 1*C*). Similarly, the frequency of IFN-γ^+^ effector OTI cells was comparable in the spleen and increased in the lymph nodes of tumor-bearing mice (Fig. 1*D*). Additionally, the proportions of IFN-γ^+^IL-2^+^ and IFN-γ^+^TNFα^+^ polyfunctional OTI effectors were similar between B16OVA-bearing and tumor-naive groups (*SI Appendix*, Fig. S5 *A* and *B*).

In summary, Tim-3-targeted vaccines, such as anti–Tim-3OTIp, induced robust antigen-specific CD8 T cell responses in tumor-bearing mice—comparable to or exceeding those in tumor-naive controls. In contrast to the impaired cross-priming observed with cDC1-targeted anti–DEC-205–based vaccination (26, 28), Tim-3-targeted vaccination overcame tumor-mediated immunosuppression and enabled efficient cross-priming of antigen-specific CD8 T cells.

### Tim-3-Targeted OVA Vaccines Induce Antigen-Specific CD8 T Cell Responses in the Absence of cDC1s.

**Fig. 2.**
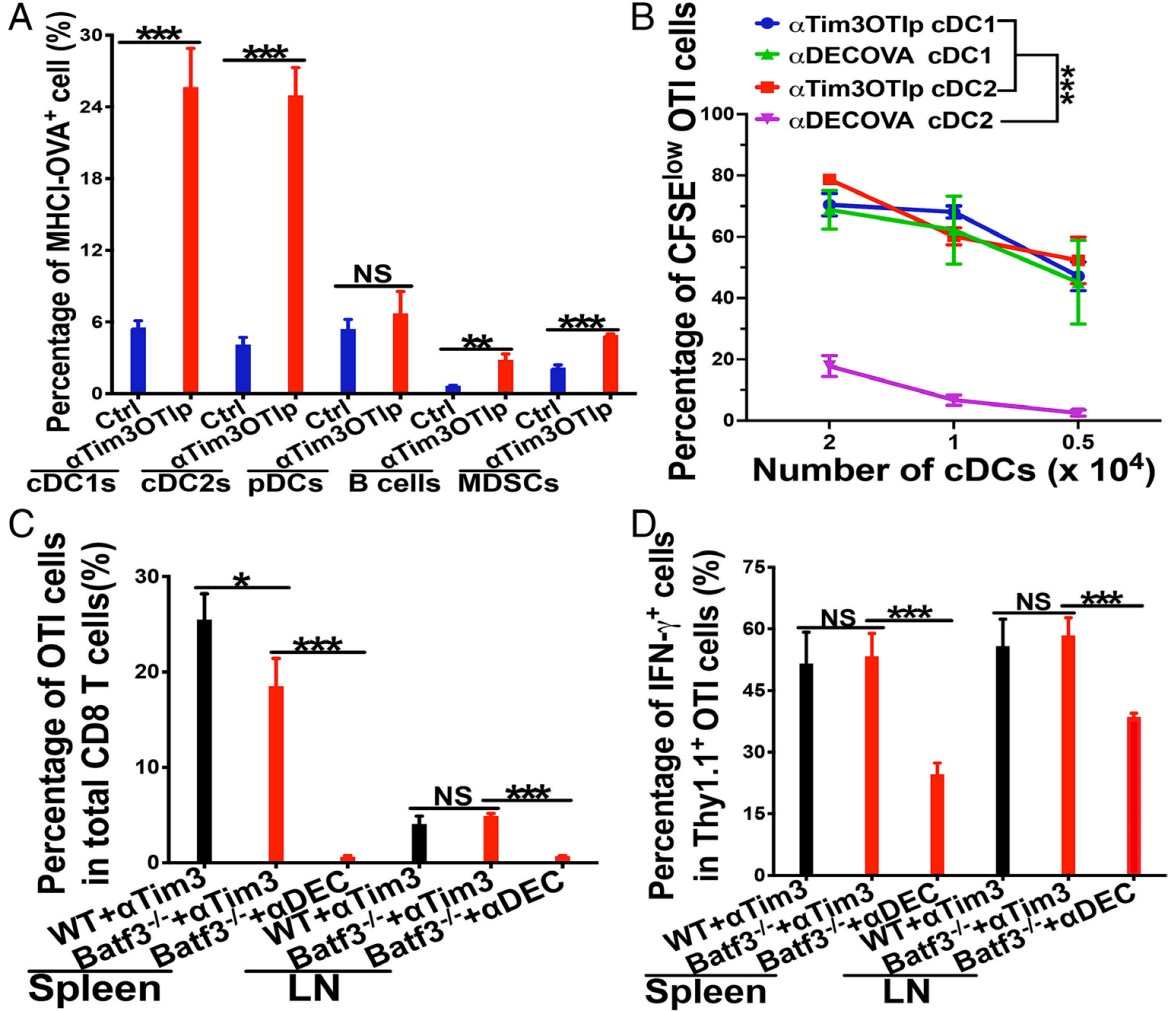
Anti-Tim-3OTIp vaccines induce cross-priming in the absence of cDC1s. (*A*) WT mice were immunized with anti-Tim-3OTIp, and the expression of MHCI-SIINFEKL (OTI epitope) complexes on various splenic APCs were examined by flow cytometry. (*B*) Splenic cDC1s or cDC2s were sorted from WT mice immunized with anti-Tim-3OTIp or anti-DEC-205OVA and cultured with naive OTI cells. The percentages of proliferated OTI cells cultured with sorted cDC1s or cDC2s as indicated were shown. (*C* and *D*) Vaccination with anti-Tim-3OTIp induced cross-priming in the absence of cDC1s. WT and Batf3^−/−^ mice (n = 5) were immunized with anti-Tim-3OTIp plus CpG and Poly I:C, with Batf3^−/−^ mice were also immunized with cDC1-targeted anti-DEC-205OVA plus CpG and Poly I:C. The percentages of Thy1.1^+^ OTI cells in total CD8 T cells (*C*), and the percentages of IFN-γ^+^ effectors out of total Thy1.1^+^ OTI cells (*D*) are shown. Student’s *t* tests for *A*, two-way mixed ANOVA for *B*, and one-way ANOVA with Bonferroni-corrected post hoc *t* tests were used for *C* and *D*. ^***^P < 0.001, ^**^P < 0.01, ^*^P < 0.05, and NS > 0.05. Data shown are representative of at least two experiments.

Our findings that Tim-3-targeted vaccines with anti-Tim-3OTIp are resistant to DC(β-catenin)- and tumor-mediated immunosuppression, together with previous data showing upregulated Tim-3 expression across multiple DC subsets in CD11c-β-catenin^active^ and tumor-bearing mice (21, 22, 29), prompted us to ask whether anti-Tim-3OTIp facilitates cross-priming through additional Tim-3-expressing APCs. Among examined APCs—including cDC1s, cDC2s, plasmacytoid DCs, B cells, and monocyte-derived suppressor cells, both cDC1s and cDC2s demonstrated appreciable cross-presentation of the OTI epitope on MHCI (H-2 K^b^-SIINFEKL complexes) following anti–Tim-3OTIp immunization (Fig. 2*A*), indicating successful acquisition, processing, and MHC I presentation by these DC subsets.

To assess the functional relevance of antigen cross-presentation in vivo, we sorted cDC1s and cDC2s from WT mice immunized with either anti-Tim-3OTIp or anti-DEC-205OVA, a benchmark cDC1-targeted vaccine known to enhance cross-presentation >1,000-fold over soluble antigen (15, 16, 30). As expected, cDC1s—but not cDC2s—from anti-DEC-205OVA-immunized mice effectively cross-primed naïve OTI cells ex vivo, inducing dose-dependent proliferation (Fig. 2*B*). In contrast, both cDC1s and cDC2s from anti-Tim-3OTIp-immunized mice robustly cross-primed OTI cells, resulting in strong proliferation of naive OTI cells across doses (Fig. 2*B*). Strikingly, cDC2s from anti–Tim-3–OTIp-immunized mice generated equivalent or even slightly higher frequencies of differentiated IFN-γ^+^ effectors out of total OTI cells compared to cDC1s from mice immunized with either anti-Tim-3OTIp or anti-DEC-205OVA (*SI Appendix*, Fig. S6). These ex vivo data place cDC2s as bona fide cross-presenters after anti-Tim-3 targeting and indicate that anti-Tim-3OTIp broadens cross-priming beyond the canonical cDC1 pathway, endowing cDC2s with cross-presentation capacity equivalent to—or higher than—cDC1s in vivo.

Since anti–Tim-3 can act on both DCs and CD8 T cells, we asked whether anti-Tim-3OTIp enhances cross-priming primarily through effects on DCs or directly on T cells. To this end, we established an in vitro system in which splenic cDCs were first pulsed with anti-Tim-3OTIp (0.03 µg/mL) and then cocultured with naive Thy1.1^+^ OTI cells, ensuring that only cDCs were initially exposed to the antibody–antigen conjugate (anti-Tim-3OTIp). After overnight coculture, Thy1.1^+^ OTI cells were purified and cultured without DCs with graded concentrations of anti-Tim-3OTIp. Addition of anti-Tim-3OTIp to OTI cells had no detectable effect on proliferation and only a slight increase in the percentages of IFN-γ-producing effectors at the highest concentration tested (2 µg/mL) (*SI Appendix*, Fig. S7). These data indicate that anti-Tim-3OTIp promotes CD8 T cell cross-priming largely through its effects on DCs, although a minor direct effect on T cells may contribute to improved effector differentiation under high-dose conditions.

To confirm that anti-Tim-3OTIp targets both cDC1s and cDC2s for cross-presentation, we compared its ability to confer cross-presenting capacity on each subset in vitro. Splenic cDCs were pulsed with isotypeOTIp, anti-Tim-3OTIp, anti-DEC-205OVA, and soluble OVA protein, after which cDC1s and cDC2s were sorted and cultured with CFSE-labeled naïve OTI cells. In cDC1 cultures, both anti-DEC-205OVA and anti-Tim-3OTIp induced robust OTI proliferation and differentiation into IFN-γ-producing effectors, whereas soluble OVA protein (pulsed at 1 µg/mL, >30× higher than antibody conjugates) and isotypeOTIp elicited little or no responses (*SI Appendix*, Fig. S8 *A* and *B*, *Left* panels). In contrast, within cDC2 cultures, anti-Tim-3OTIp consistently induced strong proliferation and effector differentiation, particularly at the lower DC:T cell ratio where anti-DEC-205–OVA failed to elicit a detectable response (*SI Appendix*, Fig. S8 *A* and *B*, *Right* panels). The modest cDC2 activity observed with anti-DEC-205OVA under more permissive in vitro conditions likely reflects assay-dependent differences in receptor-targeting readouts as previously reported (36). Collectively, the ex vivo and in vitro data converge on a clear distinction: while anti-DEC-205OVA primarily rely on cDC1s for cross-presentation, anti-Tim-3OTIp effectively engages both cDC1s and cDC2s, endowing cDC2s with cross-presenting function that matches or surpasses cDC1s under physiological conditions (Fig. 2*B*).

We next asked whether Tim-3-targeted vaccines could bypass cDC1s to enable cross-priming in vivo. WT and Batf3^−/−^ mice--which lack cDC1s (37), were adoptively transferred with Thy1.1^+^ OTI cells, and immunized with anti-Tim-3OTIp plus adjuvants. As control, Batf3^−/−^ mice were immunized with cDC1-targeting anti-DEC-205OVA. Remarkably, Batf3^−/−^ mice immunized with anti-Tim-3OTIp exhibited substantial OTI cell proliferation, though both the percentages of Thy1.1^+^ OTI cells and proliferating OTI cells at ≥6 divisions were slightly lower than in WT mice (Fig. 2*C* and *SI Appendix*, Fig. S9). In addition, the percentages of IFN-γ^+^Thy1.1^+^ OTI effectors were similar in WT and Batf3^−/−^ mice (Fig. 2*D*). In contrast, immunization of Batf3^−/−^ mice with anti-DEC-205OVA failed to induce OTI expansion in Batf3^−/−^ mice (Fig. 2*C*). These results demonstrate that anti-Tim-3OTIp is capable of inducing robust cross-priming of antigen-specific CD8 T cells in the absence of cDC1s.

Together, these results demonstrate that Tim-3-targeted vaccines can induce potent cross-priming without requiring cDC1s, likely by engaging Tim-3-expressing APCs such as cDC2s.

### Tim-3-Targeted Vaccines Against Melanoma Antigen Human gp100 Overcome DC (β-Catenin)- and Tumor-Mediated Immunosuppression and Elicit Potent Anti-Tumor Immunity.

To investigate tumor antigen-specific CD8 T cell responses, we generated an anti-Tim-3 antibody conjugated to the melanoma antigen human gp100 (anti-Tim-3hgp100). hgp100 is a homologue of mouse self/tumor antigen gp100 (mgp100) and contains the altered peptide epitope hgp100_25-33_ (KVPRNQDWL) for MHCI (H-2D^b^), which elicits gp100-specific CD8 T cell immunity in the B16 melanoma model (38–40). We first assessed whether Tim-3-targeted vaccines with anti-Tim-3hgp100 could efficiently cross-prime gp100-specific CD8 T cells. WT mice were adoptively transferred with gp100-specific Thy1.1^+^ CD8 T (Pmel1) cells, and immunized with either anti-Tim-3hgp100 or isotypehgp100 together with poly I:C and CpG as adjuvants. Immunization with anti-Tim-3hgp100 led to robust proliferation of gp100-specific CD8 T (Pmel1) cells, with significantly higher percentages of Thy1.1^+^ Pmel1 out of total CD8 T cells compared to WT mice immunized with isotypehgp100 (*SI Appendix*, Fig. S10*A*). In addition, WT mice immunized with anti-Tim-3hgp100 exhibited higher percentages of IFN-γ-producing Pmel1 effector cells out of total CD8 T cells (*SI Appendix*, Fig. S10*B*). These results are consistent with the phenotypes observed with anti-Tim-3OTIp compared to isotypeOTIp.

**Fig. 3.**
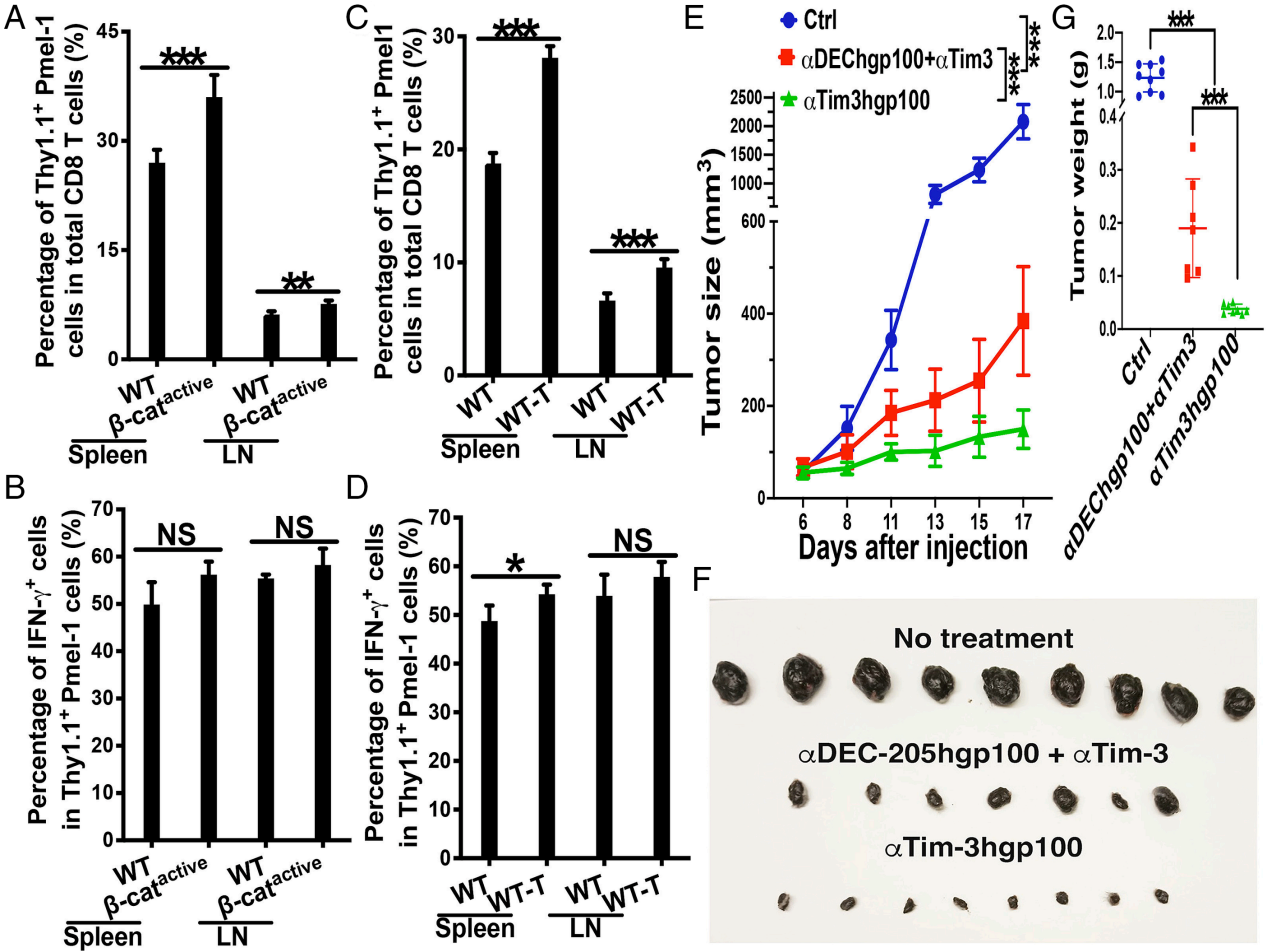
Tim-3-targeted vaccines with anti-Tim-3hgp100 overcome DC(β-catenin)- and tumor-mediated immunosuppression in inducing cross-priming. (*A* and *B*) Vaccination with anti-Tim-3hgp100 induced higher cross-priming in CD11c-β-catenin^active^ mice than WT mice. WT and CD11c-β-catenin^active^ mice (n = 4 and 5) were immunized with anti-Tim-3hgp100 plus CpG and Poly I:C. Cross-priming of adoptively transferred Thy1.1^+^ gp100-specific T (Pmel1) cells was examined at day 4 after immunization. The percentages of Thy1.1^+^ Pmel1 cells in total CD8 T cells (*A*), and the percentages of IFN-γ^+^ effectors out of total Thy1.1^+^ Pmel1 cells (*B*) are shown. (*C* and *D*) Vaccination with anti-Tim-3hgp100 induced stronger cross-priming in B16F10-bearing mice compared to tumor-naive WT mice. Tumor-naive and B16F10-bearing WT mice (n = 5) were immunized with anti-Tim-3hgp100. The percentages of Thy1.1^+^ Pmel1 cells in total CD8 T cells (*C*), and the percentages of IFN-γ^+^ effectors out of total Thy1.1^+^ Pmel1 cells (*D*) are shown. (*E*–*G*) Tim-3-targeted vaccines with anti-Tim-3hgp100 achieved better anti-tumor efficacy than cDC1-targeted DC vaccines in combination with anti-Tim-3 ICB. B16F10-bearing WT mice (n = 7 to 9) were not treated, immunized with anti-Tim-3hgp100, or with anti-DEC-205hgp100 plus anti-Tim-3 ICB (tumor sizes around 3 to 7 mm), following Thy1.1^+^Pmel-1 CD8 T cell transfer. Tumor sizes from the day of treatment are shown in (*E*), Photo of the tumors (*F*) and tumor weight (*G*) at the day 17 after tumor inoculation. Student’s *t* tests for *A*–*D*, one-way ANOVA with Bonferroni-corrected post hoc *t* tests were used for *G*, and two-way mixed ANOVA was performed to determine the difference between groups in *E*. ^***^P < 0.001, ^**^P < 0.01, ^*^P < 0.05, and NS > 0.05. Data are representative of two to four experiments.

Next, we investigated whether Tim-3-targeted vaccines using anti-Tim-3hgp100 could overcome β-catenin (DC)-mediated immunosuppression in inducing gp100-specific CD8 T (Pmel1) cell responses. WT and CD11c-β-catenin^active^ mice were adoptively transferred with Thy1.1^+^ gp100-specific CD8 T (Pmel1) cells, and immunized with anti-Tim-3hgp100 plus adjuvants as above. Similar to our findings with anti-Tim-3OTIp, CD11c-β-catenin^active^ mice immunized with anti-Tim-3hgp100 exhibited higher percentages of Thy1.1^+^ Pmel1 out of total CD8 T cells compared to WT mice (Fig. 3*A*). Furthermore, primed Pmel1 cells in CD11c-β-catenin^active^ mice exhibited comparable percentages of differentiated IFN-γ^+^ effectors compared to WT mice (Fig. 3*B*). CD11c-β-catenin^active^ mice also showed comparable percentages of IFN-γ^+^IL-2^+^ and IFN-γ^+^TNFα^+^ polyfunctional Pmel1 effector cells to WT mice (*SI Appendix*, Fig. S11 *A* and *B*). These data indicate that β-catenin in DCs does not act as a negative regulator for anti-Tim-3hgp100-induced cross-priming. Collectively, our results demonstrate that Tim-3-targeted vaccines using either the model antigen OVA or the melanoma antigen hgp100 can overcome DC(β-catenin)-mediated immunosuppression in inducing antigen-specific CD8 T cell responses.

We next evaluated the cross-priming capacity of anti–Tim-3hgp100 in B16F10 tumor-bearing mice. B16F10-bearing and tumor-naive WT mice were immunized with anti-Tim-3hgp100 plus adjuvants following adoptive transfer of Thy1.1^+^ Pmel1 cells. B16F10-bearing mice exhibited higher percentages of Thy1.1^+^ Pmel1 cells out of total CD8 T cells compared to tumor-naive WT mice (Fig. 3*C*), mirroring results obtained with anti-Tim-3OTIp. Furthermore, primed Pmel1 cells in B16F10-bearing mice displayed comparable percentages of differentiated IFN-γ^+^ effectors out of total Pmel1 cells to those in tumor-naive WT mice (Fig. 3*D*). Additionally, IFN-γ^+^IL-2^+^ and IFN-γ^+^TNFα^+^ polyfunctional Pmel1 effectors were similar or higher in B16F10-bearing mice compared to tumor-naive WT mice (*SI Appendix*, Fig. S12 *A* and *B*), reinforcing the capacity of Tim-3-targeted vaccination to overcome tumor-mediated immunosuppression.

Last, we evaluated the anti-tumor efficacy of Tim-3-targeted vaccines with anti-Tim-3hgp100. WT mice were inoculated with B16F10 melanoma cells, and were either left untreated, treated with anti-Tim-3hgp100, or anti-DEC-205hgp100 in combination with anti-Tim-3 ICB (26), together with adoptive transfer of Pmel1 cells. We selected the anti-DEC-205hgp100 plus anti-Tim-3 ICB combination as a more stringent benchmark, since our prior work showed it substantially suppresses tumor growth relative to anti-DEC-205hgp100 vaccination alone (26). Notably, B16F10-bearing WT mice treated with anti-Tim-3hgp100 resulted in even greater tumor growth inhibition than the benchmark combination of the anti-DEC-205hgp100 vaccine and anti-Tim-3 ICB (Fig. 3*E*), as demonstrated by significantly smaller tumors at endpoint (Fig. 3 *F* and *G*).

In summary, Tim-3-targeted vaccines with hgp100 induce robust tumor antigen–specific CD8 T cell responses and potent anti-tumor immunity. Notably, this platform surpasses cDC1-targeted vaccination even when combined with checkpoint inhibition, highlighting its promise as a next-generation cancer vaccine strategy.

### Tim-3-Targeted Vaccines with Anti-Tim3hgp100 Induce gp100-Specific CD8 T Cell Immunity and Anti-tumor Efficacy in the Absence of cDC1s.

**Fig. 4.**
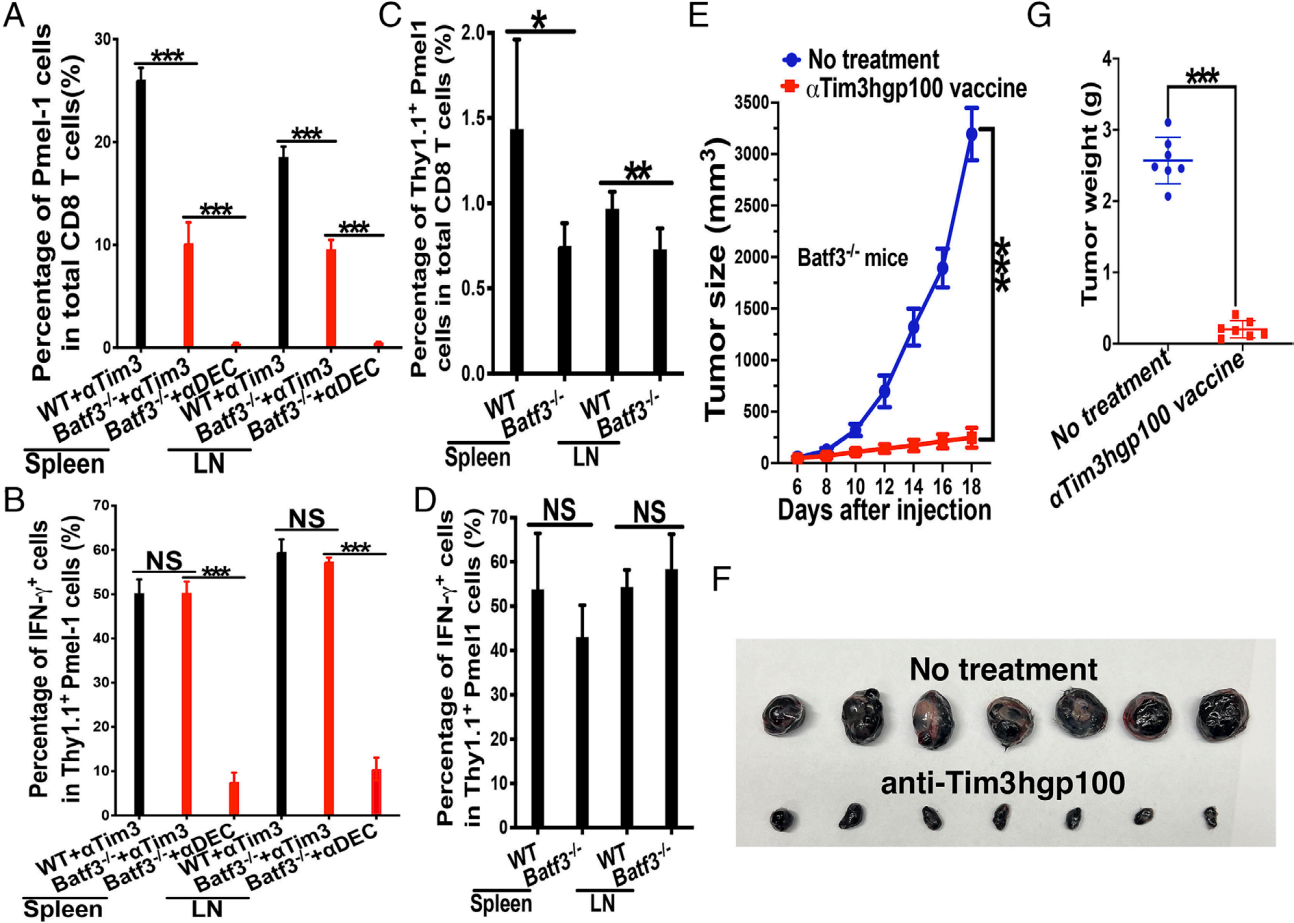
Tim-3-targeted vaccines with hgp100 (anti-Tim-3hgp100) induce potent CD8 T cell immunity and anti-tumor immunity in the absence of cDC1s. (*A* and *B*) Anti-Tim-3hgp100 induced cross-priming in the absence of cDC1s. WT and Batf3^−/−^ mice (n = 5) were immunized with anti-Tim-3hgp100, with Batf3^−/−^ mice were also immunized with cDC1-targeted anti-DEC-205hgp100. Cross-priming of adoptively transferred Thy1.1^+^ gp100-specific T (Pmel1) cells was examined at day 4 after immunization. The percentages of Thy1.1^+^ Pmel1 cells in total CD8 T cells (*A*), and the percentages of IFN-γ^+^ effectors out of total Thy1.1^+^ Pmel1 cells (*B*) are shown. (*C* and *D*) Vaccination with anti-Tim-3hgp100 generated memory gp100-specific 8 T cell responses. WT and Batf3^−/−^ mice (n = 5) immunized with anti-Tim-3hgp100 were recalled at day 20 following immunization. The percentages of Thy1.1^+^ Pmel1 cells in total CD8 T cells (*C*) and the percentages of IFN-γ^+^ effectors out of total Thy1.1^+^ Pmel1 cells (*D*) are shown. (*E*–*G*) Tim-3-targeted vaccines with anti-Tim-3hgp100 achieve anti-tumor efficacy without cDC1s. B16F10-bearing Batf3^−/−^ mice (n = 7) were not treated, or immunized with anti-Tim-3hgp100 when tumor sizes were around 3 to 6 mm. Tumor sizes (*E*), photo of the tumors (*F*), and tumor weight (*G*) at the end of experiments are shown. One-way ANOVA with Bonferroni-corrected post hoc *t* tests were used for *A* and *B*, Student’s *t* tests for *C*, *D*, and *G*, and two-way mixed ANOVA was performed to determine the difference between groups in *E*. ^***^P < 0.001, ^**^P < 0.01, ^*^P < 0.05, and NS > 0.05. Data are representative of at least two experiments.

We next investigated whether Tim-3hgp100 vaccination could generate gp100-specific CD8 T (Pmel1) cell immunity in the absence of cDC1s. WT and Batf3^−/−^ mice adoptively transferred with Thy1.1^+^ Pmel1 cells were immunized with anti-Tim-3hgp100 plus adjuvants. As a negative control, Batf3^−/−^ mice were immunized with cDC1-targeted anti-DEC-205hgp100 [anti-DEC-205 engineered to express hgp100 (26)]. As expected, Batf3^−/−^ mice immunized with anti-DEC-205hgp100 exhibited minimal expansion of Pmel1 cells (Fig. 4*A*). Consistent with our observation with anti-Tim-3OTIp, immunization with anti-Tim-3hgp100 led to robust proliferation/expansion of Pmel1 cells in Batf3^−/−^ mice, although the percentages were lower than that of WT mice (Fig. 4*A*). Further analysis revealed that primed Pmel1 cells in WT and Batf3^−/−^ mice exhibited similar percentages of differentiated IFN-γ^+^ effectors out of total Pmel1 cells (Fig. 4*B*), indicating that anti-Tim-3hgp100 can cross-prime gp100-specific CD8 T (Pmel1) cells in the absence of cDC1s.

We next assessed whether immunization with anti-Tim3hgp100 generated gp100-specific CD8 T cell memory responses without cDC1s. WT and Batf3^−/−^ mice were immunized as above and then challenged at day 21 with human gp100 epitope (gp100_25-33_) to assess memory CD8 T cell responses. Consistent with our cross-priming data, Batf3^−/−^ mice exhibited memory responses of gp100-specific CD8 T cells, although the percentages of Thy1.1^+^ Pmel1 cells were lower than that of WT mice (Fig. 4*C*). Furthermore, recalled Pmel1 cells in Batf3^−/−^ mice exhibited comparable percentages of IFN-γ^+^-producing Pmel-1 cells compared to Pmel1 cells in WT mice (Fig. 4*D*). Taken together, these data demonstrate that anti-Tim-3hgp100 is capable of generating gp100-specific memory CD8 T cell responses in the absence of cDC1s.

Finally, we examined whether anti-Tim-3hgp100 vaccine could achieve anti-tumor efficacy in the absence of cDC1s. B16F10-bearing Batf3^−/−^ mice were either untreated, or treated with anti-Tim-3hgp100 together with adoptive transfer of Pmel1 cells. As shown in Fig. 4 *E*–*G*, anti-Tim-3hgp100-treated Batf3^−/−^ mice exhibited substantially slower tumor growth compared to untreated B16F10-bearing Batf3^−/−^ mice, resulting in significantly smaller tumor sizes.

Taken together, our data using anti-Tim-3hgp100 further confirmed that anti-Tim-3-based vaccines induce effective tumor antigen-specific CD8 T cell responses and anti-tumor efficacy, even in the absence of cDC1s.

### Anti-Tim-3 Neoantigen Vaccines Eradicate Existing Solid Tumors and Generate Protective Memory Responses.

**Fig. 5.**
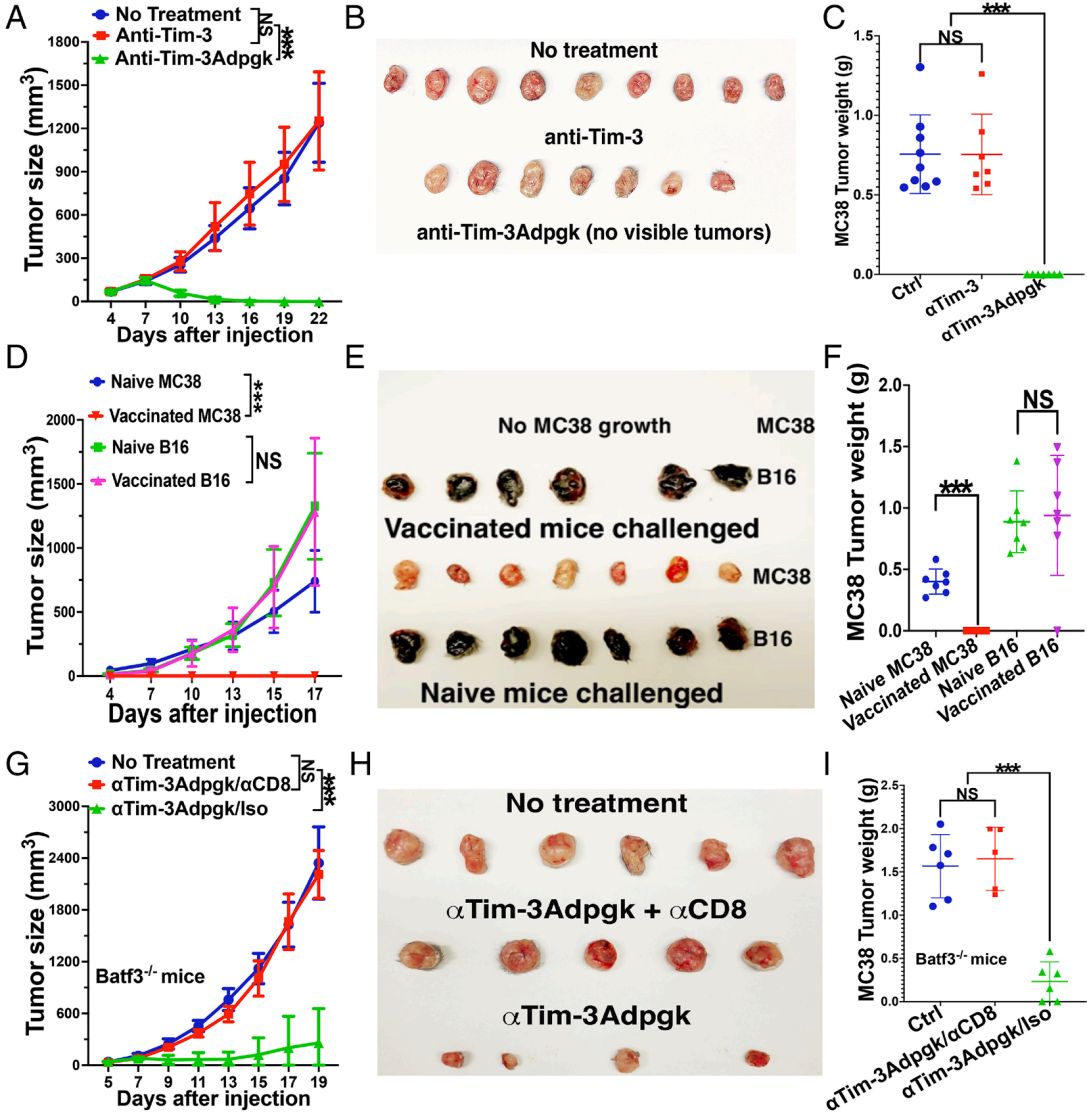
Tim-3-targeted vaccines with MC38 neoantigen Adpgk_299-307_ (anti-Tim-3Adpgk) eradicate existing tumors and generate protective memory responses. (*A*–*C*) Vaccination with anti-Tim-3Adpgk completely eradicated large MC38 tumors. MC38-bearing WT mice (n = 7 to 9) were not treated, treated with anti-Tim-3 ICB (300 µg × 2), or immunized with anti-Tim-3Adpgk (40 µg) when tumors are about 5 to 9 mm (at day 7). Tumor sizes (*A*), photo of the tumors (*B*), and tumor weight (*C*) at the end of experiments are shown. (*D*–*F*) Anti-Tim-3Adpgk vaccines generate protective antigen-specific immunity. Anti-Tim-3Adpgk-treated MC38-bearing WT mice that eradicated the tumors and naive WT mice (n = 7) were challenged with MC38 and B16F10. Tumor sizes of MC38 and B16F10 tumors (*D*), photo of the tumors (*E*) and tumor weight (*F*) at the end of experiments are shown. (*G*–*I*) Vaccination with anti-Tim-3Adpgk requires CD8 T cells but not cDC1s to achieve anti-tumor efficacy. MC38-bearing Batf3^−/−^ mice (n = 5 to 6) were not treated, or immunized with anti-Tim-3Adpgk (40 µg) with either anti-CD8 depleting antibody (anti-Tim-3Adpgk/anti-CD8) or isotype control antibody (anti-Tim-3Adpgk/Iso) when tumors are about 5 to 9 mm. Tumor sizes (*G*), photo of the tumors (*H*), and tumor weight (*I*) at the end of experiments are shown. Two-way mixed ANOVA was performed to determine the difference between groups in *A*, *D*, and *G*, one-way ANOVA with Bonferroni-corrected post hoc *t* tests were used for *C* and *I*, and Student’s *t* tests for *F*. ^***^P < 0.001, and NS > 0.05. Data are representative of two experiments.

As vaccines with tumor neoantigens including DC-based vaccines have shown promising results in clinical trials (19, 41–44), we next evaluated whether anti-Tim-3-based vaccines using neoantigens could achieve robust anti-tumor efficacy. We thus selected Adpgk, a neoantigen for the colorectal tumor cell line MC38 (45), and conjugated a long peptide containing Adpgk neoantigen epitope (Adpgk_299-307:_ ASMTNM*ELM, *denote A→M mutation) to anti-Tim-3 to generate anti-Tim-3Adpgk. This vaccine relies on processing of anti-Tim-3Adpgk to allow the neoantigen epitope to be cross-presented on MHCI (H-2D^b^). Remarkably, a single vaccination with anti-Tim-3Adpgk (40 µg/mouse) led to complete eradication of large preexisting tumors (5 to 9 mm) in all MC38-bearing mice, whereas treatment with anti-Tim-3 (300 µg/mouse at day 0 and 2) had no effect on tumor growth and tumor sizes (Fig. 5 *A*–*C*).

Given the efficacy of anti-Tim-3Adpgk in controlling tumors, we next investigated whether this vaccine could generate immunological memory capable of preventing cancer recurrence. Anti-Tim-3Adpgk-treated mice that had eradicated MC38 tumors were rechallenged with MC38 and unrelated B16F10 tumor cells 20 d postvaccination. Strikingly, anti-Tim-3Adpgk-treated mice were completely protected against MC38 tumor rechallenge but not against the unrelated B16F10 tumor, with only 1 out of 7 mice exhibiting protection against B16 challenge (Fig. 5 *D*–*F*). In contrast, both MC38 and B16F10 tumors grew robustly in naïve mice (Fig. 5 *D*–*F*). These results demonstrate that anti-Tim-3 neoantigen vaccines (anti-Tim-3Adpgk) not only achieve strong anti-tumor efficacy by eradicating large tumors but also induce antigen-specific memory responses that confer protection against MC38 but not unrelated B16F10 challenge.

To assess whether anti-Tim-3Adpgk vaccines can elicit anti-tumor immunity in the absence of cDC1s, we evaluated their anti-tumor efficacy in Batf3^−/−^ mice. MC38-bearing Batf3^−/−^ mice were either untreated, or treated with anti-Tim-3Adpgk together with either a depleting anti-CD8 or isotype control antibody. Anti-Tim-3Adpgk treatment with the isotype control antibody significantly slowed tumor growth compared to untreated mice, resulting in significantly smaller tumor sizes, with 2 out of 6 mice achieving tumor eradication (Fig. 5 *G*–*I*). However, treatment with anti-Tim-3Adpgk combined with the depleting anti-CD8 antibody completely abrogated its anti-tumor efficacy, with tumor growth comparable to that of untreated mice (Fig. 5*G*). These results indicate that anti-Tim-3Adpgk vaccination can achieve anti-tumor efficacy without cDC1s but requires CD8 T cells.

To determine whether vaccine efficacy requires trafficking of newly primed T cells from lymph nodes (LNs) to tumors, we treated MC38 tumor–bearing mice with anti-Tim-3Adpgk, either alone or together with FTY720 to block S1P-dependent lymphocyte egress. Anti-Tim-3Adpgk vaccination alone eradicated established tumors, whereas coadministration of FTY720 markedly blunted tumor control (*SI Appendix*, Fig. S13*A*), indicating that LN-to-tumor T cell trafficking is critical for optimal therapeutic efficacy (*SI Appendix*, Fig. S13*A*). Nonetheless, anti-Tim-3Adpgk vaccination in the presence of FTY720 still significantly delayed tumor growth relative to untreated controls, indicating that vaccine activity is not solely dependent on newly emigrating LN T cells and may also engage preexisting intratumoral T cells and/or local T cell expansion.

We next asked whether Tim-3-targeted neoantigen vaccines could maintain anti-tumor efficacy in ICB–resistant tumors. To address this, we used the ICB-refractory Lewis lung carcinoma (LLC) model, which is also resistant to standard peptide-based neoantigen vaccines targeting mRiok1 even when combined with anti–CTLA-4 or anti–PD-1 therapy (31). To rigorously dissect the contribution of endogenous cDC1s to vaccine efficacy, LLC-bearing WT and Batf3^−/−^mice were left untreated or immunized with anti–Tim-3mRiok1 (anti–Tim-3 conjugated to a long peptide containing the mRiok1_312–319_ neoepitope), and tumor progression and endpoint burden were assessed.

Strikingly, neoantigen anti–Tim-3mRiok1 vaccination markedly inhibited LLC tumor growth compared to untreated mice in both WT and Batf3^−/−^ groups (*SI Appendix*, Fig. S14*A*), indicating that Tim-3-targeted neoantigen vaccines can overcome resistance to conventional neoantigen vaccination plus ICB.

Comparing vaccinated WT and Batf3^−/−^groups further clarified the role of cDC1s. Although anti–Tim-3mRiok1 vaccination achieved stronger tumor control in WT mice, it still conferred significant anti-tumor efficacy in Batf3^−/−^ mice (*SI Appendix*, Fig. S14 *A*–*C*). These results demonstrate that cDC1s are not strictly required for Tim-3-targeted vaccine-mediated tumor control, while the enhanced response in WT mice indicates that cDC1s contribute to optimal efficacy. Together with our findings in the B16 and MC38 models, these data underscore the broad and robust therapeutic potential of Tim-3-targeted vaccines across different tumor settings, including those resistant to ICB and conventional neoantigen vaccines.

### Tim-3-Targeted Vaccine Platform Enables Cross-Priming with Protein Antigens and Multiple Antigenic Epitopes in the Absence of cDC1s.

**Fig. 6.**
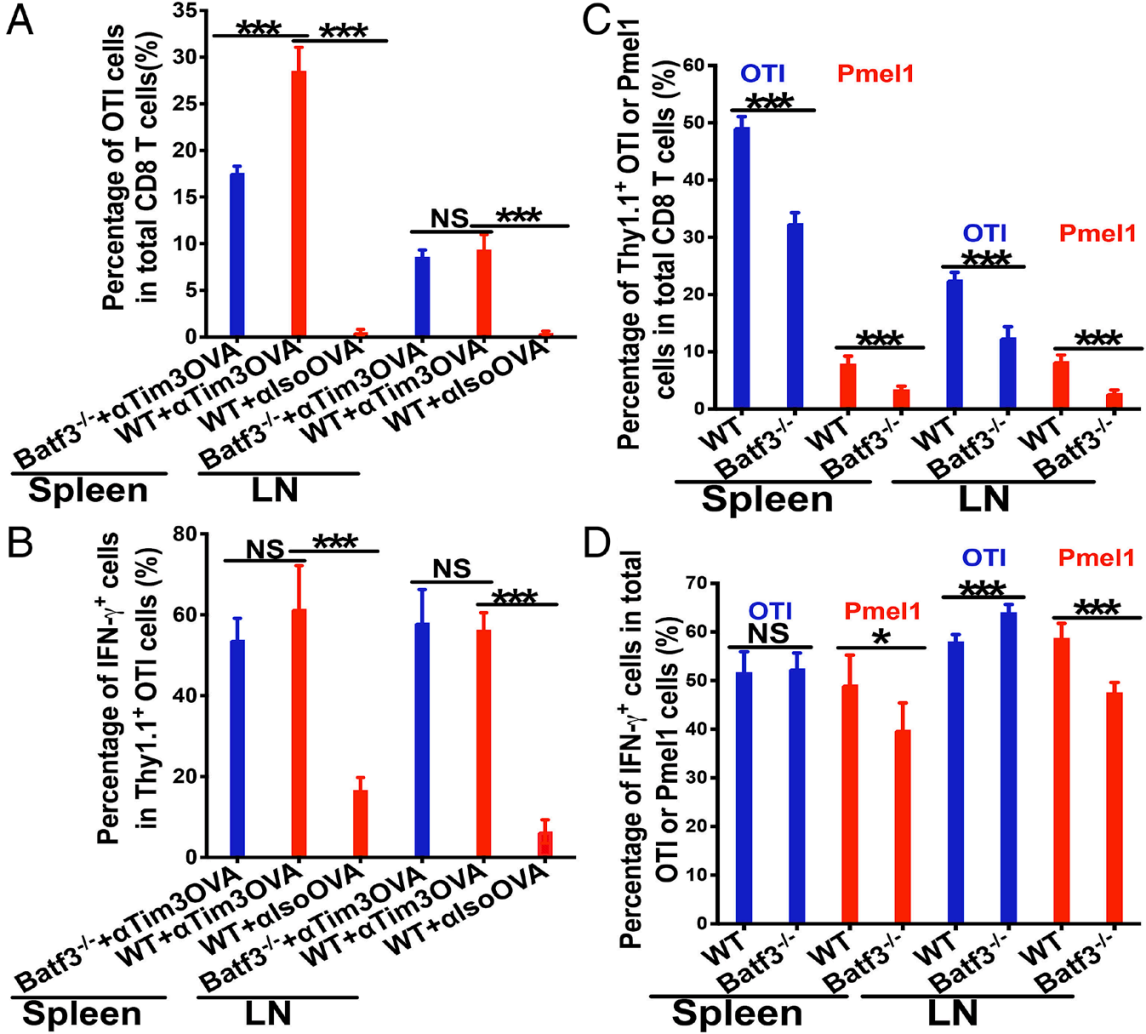
Anti-Tim-3-based vaccines with protein antigen or multiple antigen epitopes efficiently induce cross-priming of antigen-specific CD8 T cells. (*A* and *B*) Tim-3-targeted vaccines with model protein antigen chicken ovalbumin (anti-Tim-3OVA) induced cross-priming of OVA-specific CD8 T (OTI) cells in the absence of cDC1s. WT and Batf3^−/−^ mice (n = 5) were immunized with anti-Tim-3OVA or isotypeOVA (only for WT mice), and cross-priming of adoptively transferred Thy1.1^+^ OTI cells was examined at day 3 after immunization. The percentages of Thy1.1^+^ OTI in total CD8 T cells (*A*), and the percentages of IFN-γ^+^ OTI effectors out of total Thy1.1^+^ OTI cells (*B*) are shown. (*C* and *D*) Tim-3-targeted vaccines with both OVA_257-264_ and hgp100_25-33_ epitopes (anti-Tim-3OTIhgp100) cross-primed both OVA- and gp100-specific CD8 T cells in the absence of cDC1s. WT and Batf3^−/−^ mice (n = 5) were immunized with anti-Tim-3OTIphgp100 with adjuvants, and cross-priming of adoptively transferred Thy1.1^+^ OTI and Thy1.1^+^ Pmel1 CD8 T cells was examined at day 4 after immunization. The percentages of Thy1.1^+^ OTI and Thy1.1^+^ Pmel1 cells in total CD8 T cells (*C*), and the percentages of IFN-γ^+^ effectors out of total Thy1.1^+^ OTI or Thy1.1^+^ Pmel1 cells (*D*) are shown. One-way ANOVA with Bonferroni-corrected post hoc *t* tests were used for *A* and *B*, and Student’s *t* tests for *C* and *D*. ^***^P < 0.001, ^*^P < 0.05, and NS > 0.05. Data are representative of two to three experiments.

Given that protein antigens can be processed into multiple epitopes to induce broader and potentially stronger anti-tumor immune responses (46–48), we next investigated whether the anti-Tim-3 platform could be utilized for protein antigen delivery. To this aim, we conjugated OVA protein to either anti-Tim-3 or isotype control antibodies to generate anti-Tim-3OVA and isotypeOVA, respectively. WT and Batf3^−/−^ mice were adoptively transferred with Thy1.1^+^ OTI cells, and immunized with either anti-Tim-3OVA or isotypeOVA (WT mice only). Both WT and Batf3^−/−^ mice immunized with anti-Tim-3OVA exhibited strong proliferation of OTI cells, resulting in high percentages of Thy1.1^+^ OTI out of total CD8 T cells compared to minimal proliferation/expansion observed with isotypeOVA immunization (Fig. 6*A*). Although OTI frequencies were modestly reduced in Batf3^−/−^ mice, both WT and Batf3^−/−^ groups immunized with anti–Tim-3OVA generated comparable frequencies of differentiated IFN-γ^+^ OTI effectors out of total OTI cells (Fig. 6*B*). These findings demonstrate that anti-Tim-3-protein antigen efficiently cross-prime antigen-specific CD8 T cells, a function that could be achieved without cDC1s.

We next asked whether anti-Tim-3 could be employed to deliver multiple antigens which may improve protection against tumor recurrence/immune escape (48, 49). To this end, we generated an anti-Tim-3 conjugate containing two well-defined MHCI epitopes (OTI_257-264_ and hgp100_25-33_)—anti-Tim-3OTIhgp100. WT and Batf3^−/−^ mice were adoptively transferred with Thy1.1^+^ OTI and Thy1.1^+^ Pmel1 CD8 T cells, and immunized with anti-Tim-3OTIhgp100 together with adjuvants. Immunization with anti-Tim-3OTIhgp100 induced robust proliferation of both OTI and Pmel1 cells in WT and Batf3^−/−^ mice, resulting in high percentages of OTI or Pmel-1 cells out of total CD8 T cells, though the percentages were slightly lower in Batf3^−/−^ mice (Fig. 6*C*). Importantly, the percentages of IFN-γ-producing effector cells out of total OTI or Pmel1 CD8 T cells were comparable between WT and Batf3^−/−^ mice (Fig. 6*D*). Together, these results provide functional proof-of-concept that Tim-3-targeted vaccines can deliver protein antigens and multiple antigenic epitopes to efficiently cross-prime CD8 T cells—even in the absence of cDC1s.

## Discussion

Here, we demonstrate that Tim-3-targeted vaccines, generated by coupling antigens to anti–Tim-3 antibodies, efficiently deliver antigens to Tim-3-expressing APCs beyond cDC1s, including cDC2s. Tim-3-targeted vaccines 1) resist both DC (β-catenin)- and tumor-mediated immunosuppression, and 2) elicit robust anti-tumor CD8 T cell immunity and anti-tumor efficacy even when cDC1s are absent. In the B16 melanoma model, anti-Tim-3hgp100 vaccination achieved stronger anti-tumor efficacy than the stringent benchmark of cDC1-targeted anti-DEC-205hgp100 vaccine combined with Tim-3 blockade (Fig. 4). In the MC38 colorectal tumor model, a single dose of anti-Tim-3Adpgk neoantigen vaccine eradicated large established tumors in all treated mice. The anti-tumor efficacy is CD8 T cell–dependent but does not strictly require cDC1s (Fig. 5). In the ICB-refractory LLC model in which peptide-based neoantigen vaccination fails to control tumor even in combination with anti–CTLA-4 or anti–PD-1 therapy (31), anti-Tim-3-based neoantigen vaccine elicits strong anti-tumor efficacy in both WT and Batf3^−/−^ mice. Taken together, these findings position Tim-3-targeted vaccines as a next-generation platform that extends APC engagement beyond cDC1s and counteracts tumor- and DC-imposed suppression of cross-priming to drive potent CD8 T cell–mediated anti-tumor immunity. The consistent anti-tumor efficacy across B16, MC38, and ICB-refractory LLC models highlights the breadth of this approach, even in settings where conventional DC-based vaccines or ICB therapies are ineffective.

ICB immunotherapies such as anti-PD-1/anti-PD-L1 and anti-CTLA-4 have revolutionized the treatment of advanced cancers, however, response rates are low even for combination ICB therapies and side effects are common (50–52). Tim-3, a newer member of immune checkpoint molecules, was originally identified as a cell surface marker of IFN-γ-producing CD4 T helper cells and cytotoxic CD8 T cells (20), and expression of Tim-3 and PD-1 are thought to mark exhausted T cells (53). While blockade of Tim-3 combined with other ICB agents (e.g., anti–PD-1 or anti–LAG-3) improves anti-tumor immunity in preclinical models, multiple clinical trials have not delivered the expected therapeutic benefit (54–56). Although initially thought to act solely through T cells, Tim-3 is also expressed on other immune cells including NK cells and DCs (57, 58), and recent studies show that Tim-3 on DCs—in particular cDC1s—plays an essential role in the anti-tumor effects of Tim-3 blockade (21, 24, 25).

While tumor-associated cDC1s express elevated Tim-3, other DC subsets including cDC2s also exhibit increased Tim-3 expression in various tumor models (22, 27), suggesting that multiple DC populations may respond to Tim-3-targeted interventions. In support of this, we found that Tim-3 expression was upregulated on both splenic cDC1s and cDC2s in B16 tumor-bearing mice (*SI Appendix*, Fig. S4), indicating that Tim-3 upregulation extends systemically beyond the TME. This is particularly relevant for systemically administered Tim-3-targeted vaccines, which rely on peripheral DCs for effective T cell priming. Moreover, DCs with active β-catenin—mimicking the tumor-induced β-catenin activation observed in DCs (28)—exhibited increased Tim-3 expression across several splenic DC subsets, including cDC1 and cDC2s (29), further supporting the idea that Tim-3-targeted strategies could engage multiple DC subsets beyond cDC1s.

In vivo DC-targeted antigen delivery via anti–DEC-205 conjugates enhances cross-presentation by over 1,000-fold compared to immunization with noncoupled soluble antigens, such as peptide-based neoantigen vaccines (15, 16, 30). Although clinical trials using human anti–DEC-205–NY-ESO-1 have shown promise (17, 59, 60), their dependence on cDC1s remains a critical limitation. In contrast, cDC2s are more abundant in both lymphoid organs and the TME, and upregulate Tim-3 under tumor-bearing conditions (22, 27, 61). While cDC1s are well known for their superior cross-presenting ability (21, 26, 62), emerging evidence suggests that other DC subsets, particularly cDC2s, may acquire cross-presenting capabilities under specific inflammatory or tumor conditions (63–66). In human cancers, high frequencies of Tim-3^+^ cDC2s are associated with poor prognosis and reduced responsiveness to neoadjuvant anti-PD-1 therapy, and they suppress tissue-resident memory CD8 T cell (CD8 TRM) responses via galectin-9–Tim-3 signaling (27), raising the possibility that Tim-3^+^ cDC2s could be harnessed or reprogrammed for therapeutic benefit. Indeed, recent evidence shows that anti–Tim-3 treatment enhances cDC2-mediated antigen presentation and improves the efficacy of combination immunotherapies (67), demonstrating that cDC2s could be harnessed to improve anti-tumor efficacy. Together, these findings support a rationale for Tim-3-targeted vaccination as a strategy to engage cDC2s and other APCs, reduce cDC1 dependence, and improve CD8 T cell priming, particularly in immunosuppressive or ICB-resistant tumor settings.

Using CD11c-β-catenin^active^ mice, a model of tumor-associated DC dysfunction driven by β-catenin activation, we observed enhanced CD8 T cell cross-priming following anti–Tim-3OTIp vaccination compared to WT mice. This contrasts with our previous finding that cDC1-targeted vaccination with anti-DEC-205OVA resulted in reduced cross-priming in CD11c-β-catenin^active^ mice relative to WT controls (28). Together, these observations support the notion that Tim-3-targeted vaccination can overcome β-catenin–driven DC dysfunction in settings where DEC-205-targeted vaccination is impaired. However, a fully powered same-cohort comparison will be required in future work to definitively establish the relative efficacy of these approaches. Notably, cross-priming was even higher in B16-bearing WT mice relative to tumor-naive controls, indicating that anti-Tim-3OTIp-based vaccines can overcome both DC (β-catenin)- and tumor-mediated immunosuppression. Consistent with this, our data indicate that Tim-3-targeted vaccination promotes antigen cross-presentation by both cDC1s and cDC2s: MHCI–OTI peptide complexes were detected on each subset, and ex vivo and in vitro assays demonstrated that both subsets efficiently cross-prime CD8 T cells (Fig. 2 and *SI Appendix*, Figs. S6 and S8). Notably, cDC2s isolated from anti–Tim-3OTIp-vaccinated mice reached cross-presentation levels comparable to, and in some settings exceeding, cDC1s targeted by the benchmark anti–DEC-205 approach, supporting the idea that Tim-3-targeted vaccination can confer robust cross-presenting capacity on cDC2s (Fig. 2 and *SI Appendix*, Figs. S6 and S8). The substantial cross-priming retained in Batf3^−/−^ hosts (Figs. 2 and 4) further supports a meaningful contribution from cDC2s and other Tim-3^+^ APCs in mediating vaccine-induced immunity. Therefore, by engaging both cDC1s and cDC2s (and potentially other Tim-3^+^ APCs) for cross-presentation (Fig. 2*B* and *SI Appendix*, Figs. S6 and S8), Tim-3-targeted vaccines may provide a functional advantage over approaches that rely predominantly on cDC1s. Taken together, these results support a model in which Tim-3-targeted vaccines expand APC engagement and help overcome tumor- and DC-mediated immunosuppression to sustain effective cross-priming and CD8 T cell immunity.

One plausible explanation for the enhanced—rather than diminished—cross-priming in CD11c-β-catenin^active^ and tumor-bearing settings is the increased Tim-3 expression on DCs under these conditions [(22, 26, 27, 29), and *SI Appendix*, Fig. S4]. Elevated Tim-3 likely increases binding and endocytic uptake of anti–Tim-3–antigen conjugates, thereby boosting antigen cross-presentation by both cDC1s and cDC2s. In parallel, anti–Tim-3 antibodies have been reported to relieve inhibitory Tim-3 signaling and restore the function of Tim-3^+^ DCs in anti-tumor immunity (21, 24, 25). We therefore propose that anti–Tim-3-Antigen vaccines operate through dual, converging mechanisms centered on Tim-3^+^ DCs: they enhance antigen delivery to these DCs while simultaneously relieving Tim-3-mediated suppression within the same cells. This coupling of targeted antigen delivery with functional restoration may underlie the potency of Tim-3-targeted vaccine platform, enabling robust cross-priming even in settings dominated by β-catenin–driven DC dysfunction and tumor-mediated immunosuppression.

Importantly, this effect was not limited to model antigens. In the ICB-insensitive B16 melanoma model, vaccination with anti–Tim-3hgp100 induced strong gp100-specific CD8 T cell responses in both CD11c-β-catenin^active^ and B16-bearing mice compared to tumor-naive controls. Therapeutically, anti-Tim-3hgp100 achieved greater tumor growth control than a stringent benchmark combining cDC1-targeted anti-DEC-205hgp100 vaccine and Tim-3 ICB, and retained anti-tumor efficacy in Batf3^−/−^ mice (Figs. 3 and 4), highlighting its potential in cDC1-deficient settings. In an ICB-refractory setting, a LLC neoantigen vaccine with anti-Tim-3mRiok1 achieved significant anti-tumor efficacy in both WT and Batf3^−/−^ mice, contrasting with prior studies in which mRiok1 peptide vaccination failed even in combination with anti–CTLA-4 or anti–PD-1 therapy (31).

We further extended these findings to another clinically relevant neoantigen in the MC38 colorectal tumor model. A single dose of anti-Tim-3Adpgk eradicated large established MC38 tumors in all treated mice, outperforming ICB therapies, DC vaccines, and combination therapies reported in this model (22, 32, 33). Mice that cleared MC38 tumors exhibited antigen-specific memory, as evidenced by complete protection upon MC38 rechallenge but not against irrelevant B16 tumors (Fig. 5). Interestingly, one mouse resisted both MC38 and B16 tumors, raising the possibility of epitope spreading—a phenomenon previously observed with neoantigen vaccines (43, 44, 68, 69). While larger studies are needed to confirm this, these results suggest that Tim-3-targeted vaccines may induce systemic immunity beyond the vaccine antigens.

Consistent with our findings with anti-Tim-3hgp100, anti-Tim-3Adpgk or anti-Tim-3mRiok1 neoantigen vaccination also conferred significant anti-tumor efficacy in Batf3^−/−^ mice, albeit not as efficient as in WT mice, suggesting cDC1s enhance optimal responses but are not strictly required. These findings suggest that Tim-3-targeted vaccines do not rely exclusively on cDC1s, but instead engage both cDC1s and other Tim-3^+^ APCs—such as cDC2s—which may act cooperatively to elicit optimal immune responses. Importantly, the therapeutic effect was dependent on CD8 T cells (Fig. 5), indicating that efficient cross-presentation and activation of neoantigen-specific CD8 T cells are critical for vaccine efficacy. Together, these results position Tim-3-targeted vaccines as a broadly applicable platform that mitigates cDC1 dependence and elicits potent CD8 T cell immunity in cancer.

The mechanisms by which Tim-3-targeted vaccines enhance cross-presentation remain incompletely understood. In human cDC2s, different receptors are known to direct antigens through distinct intracellular pathways, affecting their function in cross-presentation (70). Among the four Tim-3 ligands, binding of phosphatidylserine has been shown to promote cross-presentation by Tim-3^+^ DCs (71), whereas HMGB1 binding inhibits nucleic acid entry into DC endosomes (24). Antigen conjugation to anti–Tim-3 may facilitate internalization into compartments that favor cross-presentation, though the intracellular trafficking routes and processing steps remain to be characterized. In parallel, Tim-3 blockade has also been shown to upregulate CXCL9 and activate inflammasomes in cDC1s (21, 25), which may synergize with antigen delivery to improve cross-presentation. Interestingly, cDC1s and cDC2s appear to regulate Tim-3 through distinct mechanisms (72), suggesting that subset-specific targeting strategies could further enhance the efficacy of Tim-3-targeted vaccines.

An alternative explanation for cDC2-mediated cross-priming is cross-dressing, in which DCs acquire preformed tumor-derived peptide–MHCI complexes (cross-dressed) in certain settings (65, 73). However, several features of our data argue against cross-dressing being the primary mechanism in the setting of Tim-3-targeted vaccination. First, cross-presentation by sorted cDC1s and cDC2s was observed after a short antigen pulse in vitro, a condition that minimizes cell-to-cell transfer and argues for intrinsic processing and cross-presentation by cDC2s. Second, both anti-Tim-3OTIp andanti-Tim-3hgp100 elicited strong antigen-specific CD8 T cell cross-priming in Batf3^−/−^ mice lacking cDC1s, demonstrating that cDC2s (and potentially other Tim-3^+^ APCs) can mediate cross-priming in the absence of cDC1s. Together, these results support direct antigen uptake and processing by cDC2s as the primary basis for their Tim-3-enhanced cross-presenting activity.

Our data highlight cDC2s as a key APC subset capable of mediating cross-priming after Tim-3-targeted vaccination, thereby expanding the functional landscape of DC-based immunotherapy beyond cDC1s. While we focused on cDC2s, other Tim-3^+^ APCs—such as transitional DCs or B cells—may also contribute, offering additional avenues for therapeutic enhancement. The specific cDC2 subsets most responsive to Tim-3-targeted delivery, and the signals required to fully activate their cross-presenting potential, remain to be elucidated. Nevertheless, our findings in both B16 and MC38 tumors, as well as the ICB-refractory LLC model, provide a compelling preclinical rationale for translation. Future studies should investigate whether these principles extend to human tumors—particularly in patients who do not respond to current immunotherapies—and how Tim-3-targeted vaccines can be refined for broader clinical application.

In conclusion, Tim-3-targeted vaccines offer a promising strategy to reduce reliance on cDC1s by engaging both cDC1s and cDC2s, and potentially other Tim-3^+^ APCs, for cross-priming. Across melanoma (B16), colorectal cancer (MC38), and ICB-refractory lung carcinoma (LLC) models, Tim-3-targeted vaccines improved tumor control. Notably, this activity was preserved in various settings where cDC1 function is limited, including established tumor-bearing hosts, DC-intrinsic dysfunction driven by β-catenin activation (CD11c-β-catenin^active^ mice), and cDC1 deficiency (Batf3^−/−^ mice). The ability of Tim-3-targeted vaccine platform to deliver diverse tumor antigens and neoantigens to DCs within a single vaccine formulation underscores its flexibility and translational potential. Together, these findings support further preclinical and translational development of Tim-3-targeted vaccines, particularly for patients whose tumors respond poorly to current immunotherapies.

## Methods

CD11c-β-catenin^active^ (CD11c-Cre^+^β-catenin^Exon3/Exon3^) and Batf3^−/−^ mice, as well as Thy1.1^+^ Rag1^−/−^ OTI and Thy1.1^+^ Pmel1 TCR transgenic mice, were used to investigate cross-priming and anti-tumor efficacy in response to Tim-3-targeted vaccination, under protocols approved by the Institutional Animal Care and Use Committee at Henry Ford Health. For in vivo immunization, peptides or OVA protein were conjugated to anti–Tim-3 or anti–DEC-205 antibodies and administered together with CpG and poly I:C adjuvants via intravenous and subcutaneous routes, followed by analysis of primary and recall CD8 T cell responses by CFSE dilution, surface phenotyping, and intracellular cytokine staining by flow cytometry. In vivo cross-priming assays, ex vivo and in vitro cross-priming assays employing sorted splenic cDC1 and cDC2 subsets from immunized or Flt3L-treated mice cocultured with naïve Thy1.1 OTI were employed to assess proliferation and effector differentiation. Therapeutic efficacy was evaluated in multiple transplantable tumor models, including B16F10, B16OVA, MC38, and LLC, with tumor-bearing mice treated with Tim-3-targeted vaccines, anti-DEC-205-based vaccines, systemic anti–Tim-3 blockade, FTY720, and adjuvants as indicated, and tumor growth monitored longitudinally. Data were analyzed using unpaired two-tailed Student’s *t* tests and one- or two-way ANOVA with appropriate post hoc tests, with *P* < 0.05 considered statistically significant. Full experimental details, including protocols, key reagents, and resources, are provided in *SI Appendix*, *Materials and Methods*.

## Supplementary Material

Appendix 01 (PDF)

## Data Availability

All data supporting the findings of this study are available within the article and its *SI Appendix*. No additional datasets were generated or deposited in public repositories.
