# Supplementary material for: Tim-3-targeted vaccines overcome tumor immunosuppression and reduce cDC1 dependence to elicit potent anti-tumor immunity
Source: Proc Natl Acad Sci U S A. 2026 Mar 19;123(12):e2518080123. doi: 10.1073/pnas.2518080123 (PMC13012078; doi:10.1073/pnas.2518080123)
Supplement: Supplementary file 1 — Appendix 01 (PDF) [file pnas.2518080123.sapp.pdf]

Supplementary Materials

Materials and Methods
Additional References
Supplemental Figure S1-S14

Materials and Methods

Mice and treatments. CD11c-β-catenin^{active} (CD11c-Cre^{+}β-catenin$^{Exon3/Exon3}$) mice were generated and maintained as previously described (1, 2). CD8 TCR transgenic Thy1.1^{+} Rag1$^{-/-}$ OTI mice with Thy1.1 congenic marker were backcrossed >20 generations to recombination activating gene 1 (Rag1)-deficient mice (3). CD8 TCR transgenic Thy1.1^{+} Pmel1 (gp100-specific CD8 T cells) mice and Batf3$^{-/-}$ mice were purchased from The Jackson Laboratory. Primary and recall responses were examined as previously described (1). For vaccinations, mice received intravenous (i.v.) injections of the following antibodies: 1-20 μg anti-Tim-3OTIp, 5-40 μg anti-Tim-3hgp100, 1-5 μg anti-Tim-3OTIphgp100, 5-20 μg anti-Tim-3OVA, 1-10 μg of anti-DEC-205OVA, 5-20 μg anti-DEC-205hgp100 (4), 40 μg anti-Tim-3Adpgk, and 40 μg anti-Tim-3mRiok1, and adjuvants CpG and poly I:C (10–25 μg each) were co-administered via both i.v. and subcutaneous (s.c.) routes in equal amounts. For tumor treatment studies, adjuvants were administered subcutaneously near the tumor site and intravenously, and for B16F10 and Lewis Lung Carcinoma (LLC) treatment, vaccination with the same vaccine was administrated 4 or 5 days after the initial treatment. Anti-Tim-3 (300 μg/per mouse) were injected intraperitoneally in PBS, at day 0 and 2, either alone or together with anti-DEC-205hgp100 vaccination. Anti-CD8 depleting antibody or isotype control antibody at 200 μg/mouse were injected intraperitoneally in PBS, at day -1, 1, 3, 5 and 7 of the immunization. FTY720 (20 μg/per mouse) were injected intraperitoneally at day -1, 1, 3 and 5 of immunization. All procedures on animals were performed in accordance with protocols approved by the Institutional Animal Care and Use Committee at Henry Ford Health.

Antibodies and reagents. Antibodies to Thy1.1 (clone OX-7), TCR Vα_2 (clone B20.1), V$\beta_{5.1/5.2}$ (clone MR9.4), Vβ_{13} (clone MR12.4), CD8α (clone 53.6.7), Siglec-H (clone 551), Bst-2 (clone 927), CD11b (clone M1/70), F4/80 (clone BM8), Gr1 (clone RB6-8C5), B220 (clone RA3-6B2), CD19 (clone 1D3), CD62L (MEL-14), CD44 (IM7), CD11c (N418), MHC class II I-A^{b} (AF6-120.1), XCR1 (ZET), CD172a (SIRPa, clone P84), MHC class I H-2K^{b} (AF6-88.5), CD103 (2E7), MHCI-SIINFEKL (25-D1.16), Tim-3 (RMT3-23), CCR7 (4B12), IFN-γ (XMG1.1), TNF-α (MT6-XT22), IL-2 (JES6-5H4), and Granzyme B (QA16A01) were purchased from Biolegend Inc (San Diego, CA, USA). FTY720 was purchased from Sigma Aldrich (St. Louis, MO). Naive CD8 T cell isolation kit, and anti-Thy1.1 and anti-CD11c magnetic microbeads were purchased from Miltenyi Biotec (Auburn, CA, USA). Anti-Tim-3 (BioXcell, clone RMT3-23), Anti-CD8α (BioXcell, clone 53.6.7), and isotype control (BioXcell, Rat IgG2a clone2A3) antibodies were purchased from BioXcell (Lebanon, NH, USA). Anti-Tim-3 and isotype control antibodies were conjugated to peptide and protein as described (5): peptides containing the epitope at the N terminus and an added cysteine and

biotin at the C terminus(TRESIINFEKLEKC-Ahx-K-biotin for OTI, GATKVPRNQDWLC-Ahx-K-Biotin for hgp100, TEKSIINFEKLEEKVPRNQDWLKC-Ahx-K-biotin for OTI and hgp100, REKELASMTNMELMERKC-Ahx-K-Biotin for Adpgk, and TEESKMYQYARLEKC-Ahx-K-Biotin for mRiok1) were synthesized and purified by HPLC (Biomatik or Genescript); anti-Tim-3 or isotype control antibodies were treated with sulfosuccinimidyl 4-[*N*-maleimidomethyl]cyclohexane-1-carboxylate (sulfo-SMCC, Thermo Fisher Scientific) to generate sulfo-reactive groups in tertiary amines, purified and added to peptides for conjugation, and the conjugates were purified with protein G sepharose column. OVA protein was treated with Tris(2-carboxyethyl)phosphine (TCEP, Sigma-Aldrich) before conjugation to SMCC-treated antibodies. A Sony MA900 was used for sorting cDC1s and cDC2s from enriched $CD11c^+$ DCs from spleen cells of mice immunized with anti-Tim-3OTIp or anti-DEC-205OVA (10 µg each for i.v. and s.c.). Staining for surface and intracellular antigen expression was performed as previously described (6). In brief, cells from spleen or pooled draining LN were stimulated for 5 hours with $OTI_{257-264}$ peptide or $hgp100_{25-33}$ peptide (4 µg/ml, AnaSpec, Fremont, CA, USA) in the presence of Brefeldin A (BFA, 5 µg/ml, Biolegend), stained for cell surface protein expression and for intracytoplasmic staining antigens like IFN-γ before subjected to flow cytometry. Where indicated, OTI or Pmel1 CD8 T cells were labeled with 5-(6)-carboxyfluorescein diacetate succinimidyl diester (CFSE) and checked by flow cytometry before transfer. We used a Celesta™ (BD Biosciences, Franklin Lakes, NJ, USA) or NovoCyte Quanteon (Agilent Technology, Santa Clara, CA, USA) with subsequent analysis of data in FlowJo® (Tree Star, Ashland, OR, USA).

In vivo, ex vivo and in vitro cross-priming assays. In vivo cross-priming assays were described previously (1). Briefly, 0.2-1 × 10^6 CFSE-labeled naïve $Thy1.1^+$ OTI or Pmel1 CD8 T cells were injected intravenously by tail vein in 200 µl PBS, at 0 or 1 days after immunization, and were in vitro stimulated and evaluated by flow cytometry. For ex vivo cross-priming assay, WT mice were immunized with anti-Tim-3OTIp, and splenic cDC1s and cDC2s were sorted next day and co-cultured with 5 × 10^4 CFSE-labeled naïve $Thy1.1^+$ OTI cells for 4-5 days, and subjected to *in vitro* stimulation and flow cytometry. For in vitro cross-priming assays, splenic cDCs were isolated from Flt3L(BioXcell)-treated WT mice with a modified protocol using anti-CD11c magnetic microbeads as previously described (7). cDCs were pulsed for 4 hours with antigens including anti-Tim-3OTIp, IsotypeOTIp, anti-DEC-205OVA (at 0.03 µg/ml), or OVA protein (at 1 µg/ml), and then cDC1s and cDC2s were sorted and co-cultured with CFSE-labeled naïve $Thy1.1^+$ OTI cells for 4–5 days. In selected experiments, purified cDCs were pulsed with anti-Tim-3OTIp (0.03 µg/ml) for 4 hours, cultured with CFSE-labeled naïve $Thy1.1^+$ OTI cells (5 × 10^4), and co-cultured OTI cells were isolated the next day using anti-Thy1.1 magnetic microbeads and subsequently cultured alone or with anti–Tim-3OTIp (0.05, 2 µg/ml) before analysis.

Tumor cell lines and treatment of tumor-bearing mice. B16F10, B16OVA and Lewis Lung Carcinoma (LLC) are obtained from ATCC, and MC38 tumor cells are obtained from Genentech. Tumor cells were inoculated by subcutaneous injection. Tumors were measured every 2-3 days once they become palpable, and tumor sizes were calculated as (0.5 × short length × long $length^2$). Mice were euthanized when tumors reached 20 mm in any one dimension or when signs of illness were observed.

Statistical analysis. The statistical significance was evaluated with Excel or GraphPad Prism 10 using two-tailed unpaired two-sample student's *t*-test and ANOVA with post hoc tests (3 or more group). For tumor growth, two-way mixed ANOVA was performed to determine the difference between groups. *P* values less than 0.05 were considered significant.

References:

1. X. Liang *et al.*, beta-catenin mediates tumor-induced immunosuppression by inhibiting cross-priming of CD8(+) T cells. *J Leukoc Biol* **95**, 179-190 (2014).
2. C. Fu *et al.*, beta-Catenin in dendritic cells exerts opposite functions in cross-priming and maintenance of CD8+ T cells through regulation of IL-10. *Proc Natl Acad Sci U S A* **112**, 2823-2828 (2015).
3. Q. Li *et al.*, A central role for mTOR kinase in homeostatic proliferation induced CD8+ T cell memory and tumor immunity. *Immunity* **34**, 541-553 (2011).
4. C. Fu *et al.*, beta-Catenin in Dendritic Cells Negatively Regulates CD8 T Cell Immune Responses through the Immune Checkpoint Molecule Tim-3. *Vaccines (Basel)* **12** (2024).
5. D. Sancho *et al.*, Tumor therapy in mice via antigen targeting to a novel, DC-restricted C-type lectin. *J Clin Invest* **118**, 2098-2110 (2008).
6. A. Jiang *et al.*, Disruption of E-cadherin-mediated adhesion induces a functionally distinct pathway of dendritic cell maturation. *Immunity* **27**, 610-624 (2007).
7. C. Fu *et al.*, Plasmacytoid dendritic cells cross-prime naive CD8 T cells by transferring antigen to conventional dendritic cells through exosomes. *Proc Natl Acad Sci U S A* 10.1073/pnas.2002345117 (2020).

Figures S1 to S14

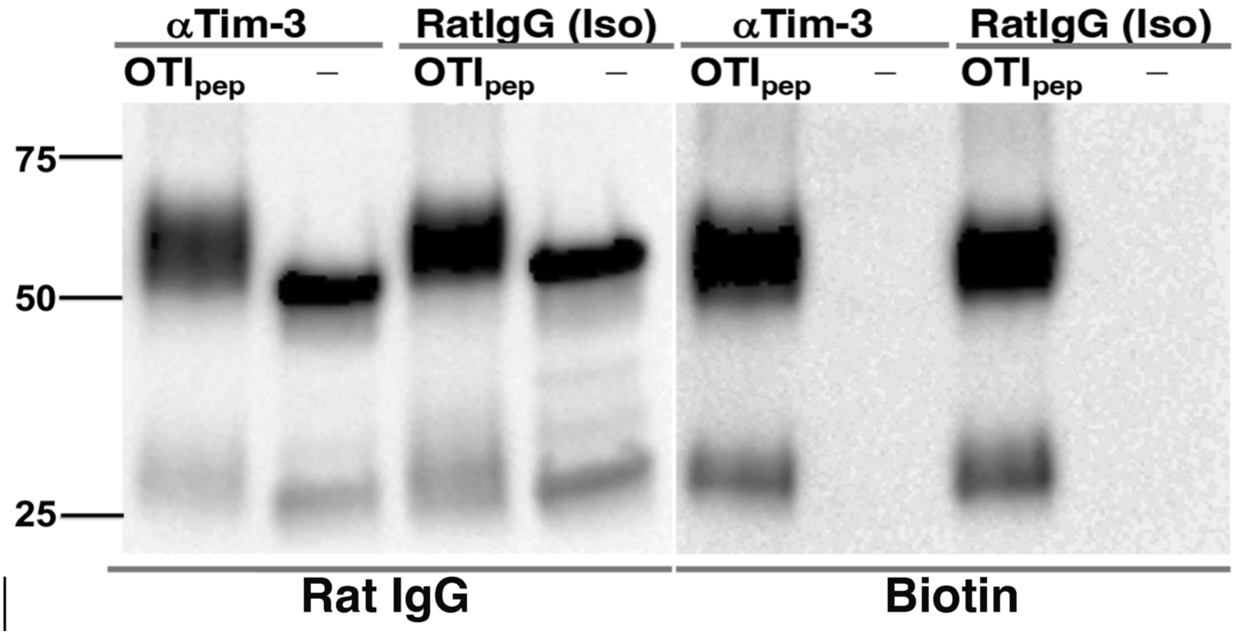

Figure S1. Generation of anti-Tim-3OTIp and isotypeOTIp by conjugating OTI-Biotin long peptide to anti-Tim-3 and isotype antibodies. Western blot for rat IgG and Biotin on anti-Tim-3 and isotype control antibodies conjugated with long peptide containing OTI epitope (OTI-Biotin). Note that anti-Biotin was not detected in unconjugated antibodies.

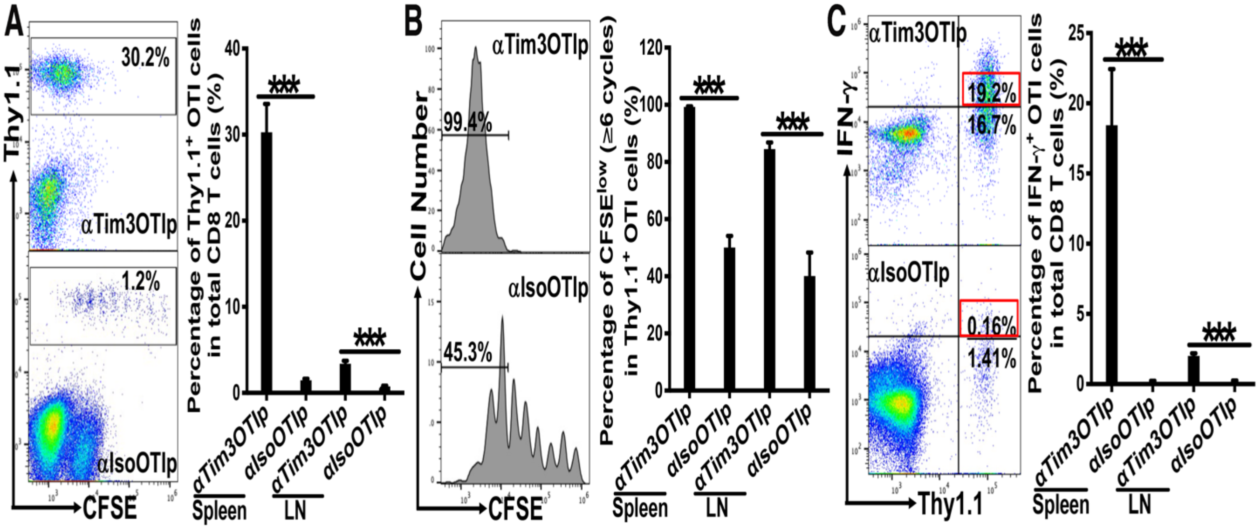

Figure S2. Tim-3-targeted vaccines with anti-Tim-3OTIp efficiently cross-prime OVA-specific CD8 T cells. WT mice (n=5) were immunized with either anti-Tim-3OTIp or isotypeOTIp with poly I:C and CpG following adoptive transfer of naïve CFSE-labeled Thy1.1$^+$ OVA-specific CD8 T (OTI) cells, and cross-priming of transferred OTI cells was examined at day 3 after immunization. **(A)** The percentages of Thy1.1$^+$ OTI cells in total CD8 T cells are shown with representative dot plots from flow cytometry (left) and bar graph (right), **(B)** the percentages of primed OTI cells that have undergone 6 or more cycles of proliferation out of total Thy1.1$^+$ OTI cells are shown with representative histogram from flow cytometry (left) and bar graph (right), **(C)** The percentages of IFN-γ^+ effectors out of total CD8 T cells following 5 hour *in vitro* stimulation with OTI$_{257\text{-}263}$ with Brefeldin A (BFA) are shown. Student's t tests, $^{***}P < 0.001$. Data are representative of two experiments.

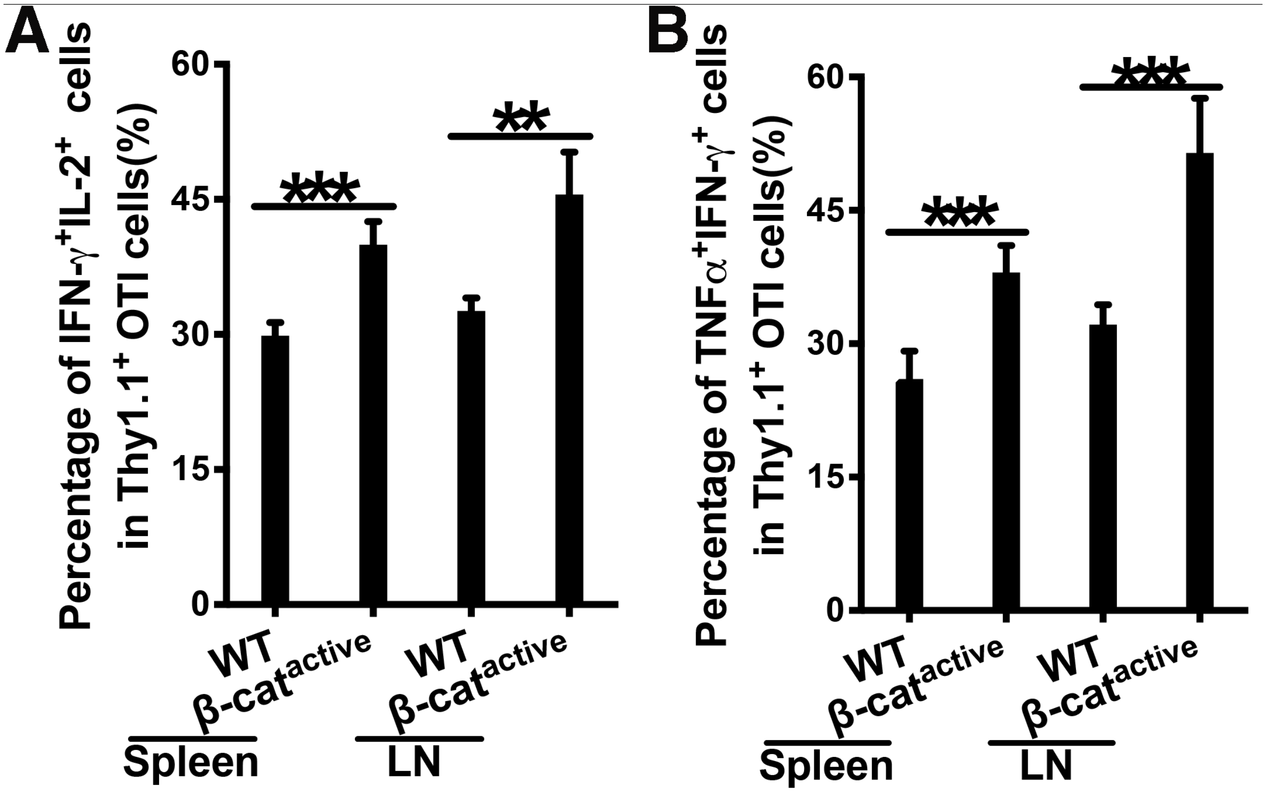

Figure S3. Anti-Tim-3OTIp vaccine-induced cross-priming is resistant to β-catenin-mediated immunosuppression. Vaccination with anti-Tim-3OTIp induced higher cross-priming in CD11c-β-catenin^{active} mice than WT mice. WT and CD11c-β-catenin^{active} mice (n=4-5) were immunized with anti-Tim-3OTIp, and cross-priming of adoptively transferred Thy1.1^{+} OVA-specific CD8 T (OTI) cells was examined at day 3 after immunization. The percentages of IFN-γ^{+}IL-2^{+} **(A)** and IFN-γ^{+}TNFα^{+} **(B)** effectors out of total Thy1.1^{+} OTI cells following 5 hour *in vitro* stimulation with OTI$_{257-263}$ with Brefeldin A (BFA) are shown. Student's t tests, $^{***}P < 0.001$, $^{**}P < 0.01$. Data are representative of two experiments.

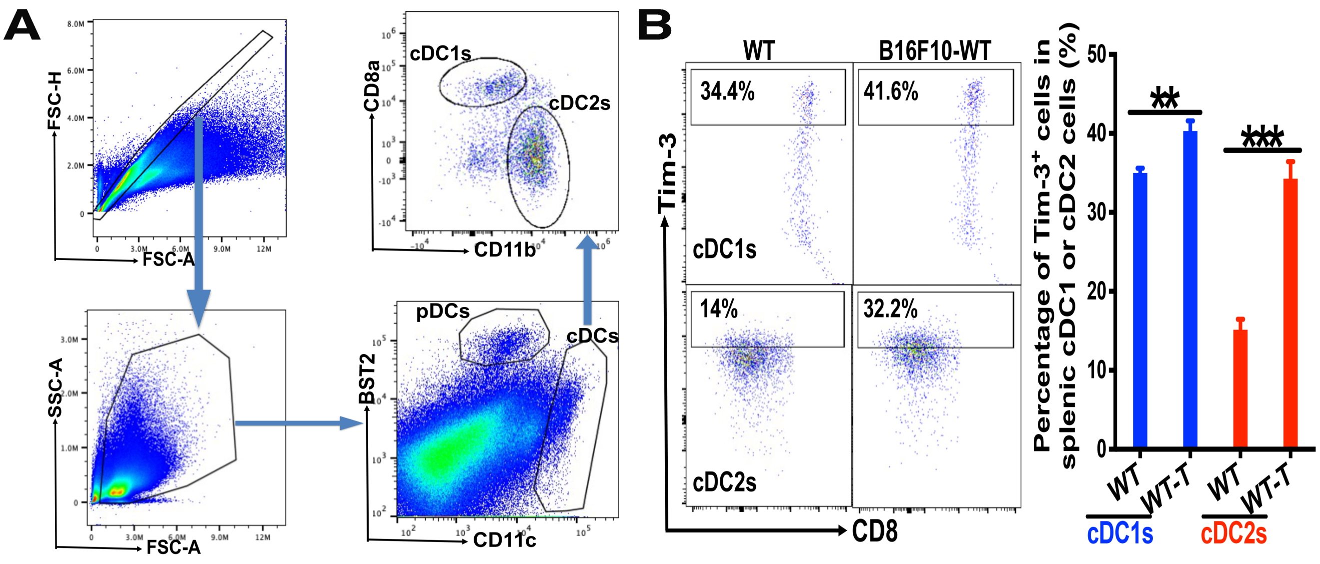

Figure S4. B16-bearing WT mice exhibited upregulated Tim-3 expression in splenic cDC1s and cDC2s. (A) Gating strategy for splenic cDCs. **(B)** B16F10-bearing WT mice upregulate Tim-3 on both splenic cDC1s and cDC2s compared to tumor-naive WT mice (n=3). Splenic cells were stained and analyzed by flow cytometry, and the percentages of Tim-3$^+$ cDC1s or cDC2s were shown with representative histogram from flow cytometry (left) and bar graph (right). Student's t tests, $^{***}P < 0.001$, $^{**}P < 0.01$. Data shown are representative of three experiments.

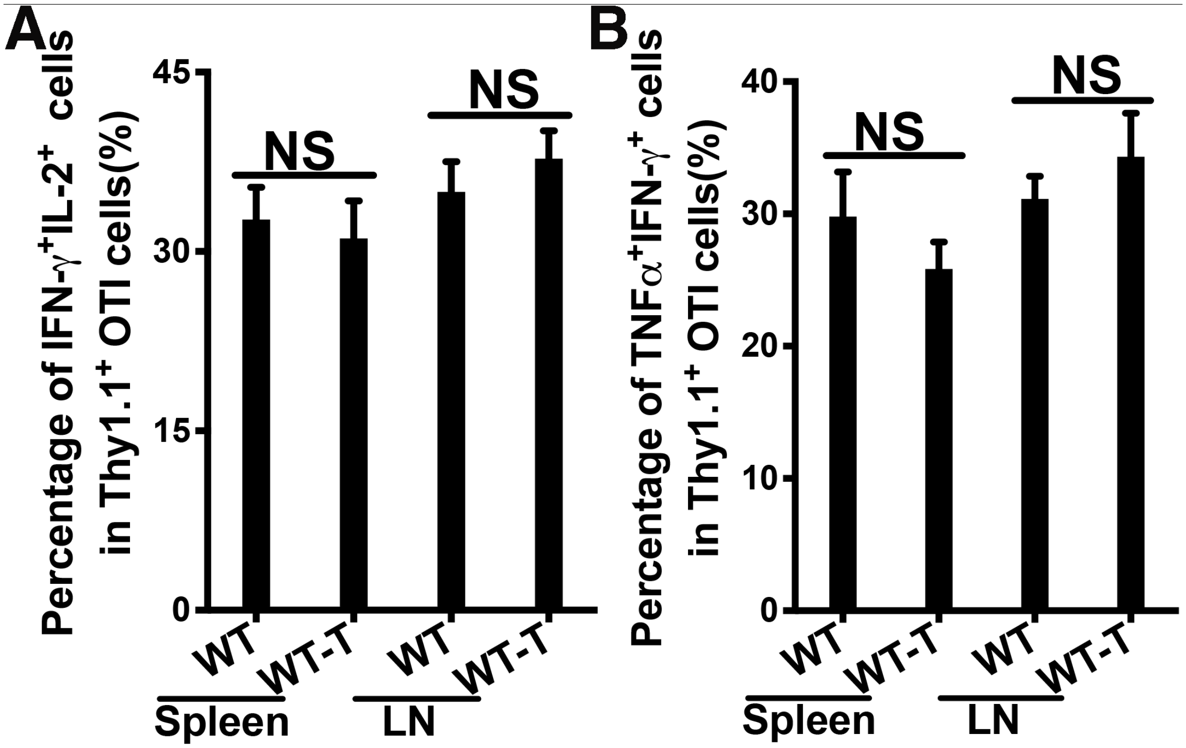

Figure S5. Anti-Tim-3OTIp vaccine-induced cross-priming is resistant to tumor-mediated immunosuppression. Vaccination with anti-Tim-3OTIp induced stronger cross-priming in B16OVA-bearing mice compared to tumor-naive WT mice. Tumor-naive and B16OVA-bearing WT mice (n=4-5) were immunized with anti-Tim-3OTIp, and cross-priming of adoptively transferred Thy1.1$^+$ OTI cells was examined at day 3 after immunization. The percentages of IFN-γ^+IL-2$^+$ **(A)** and IFN-γ^+TNFα^+ **(B)** effectors out of total Thy1.1$^+$ OTI cells following 5 hour *in vitro* stimulation with OTI$_{257-263}$ with Brefeldin A (BFA) are shown. Student's t tests, NS > 0.05. Data are representative of two experiments.

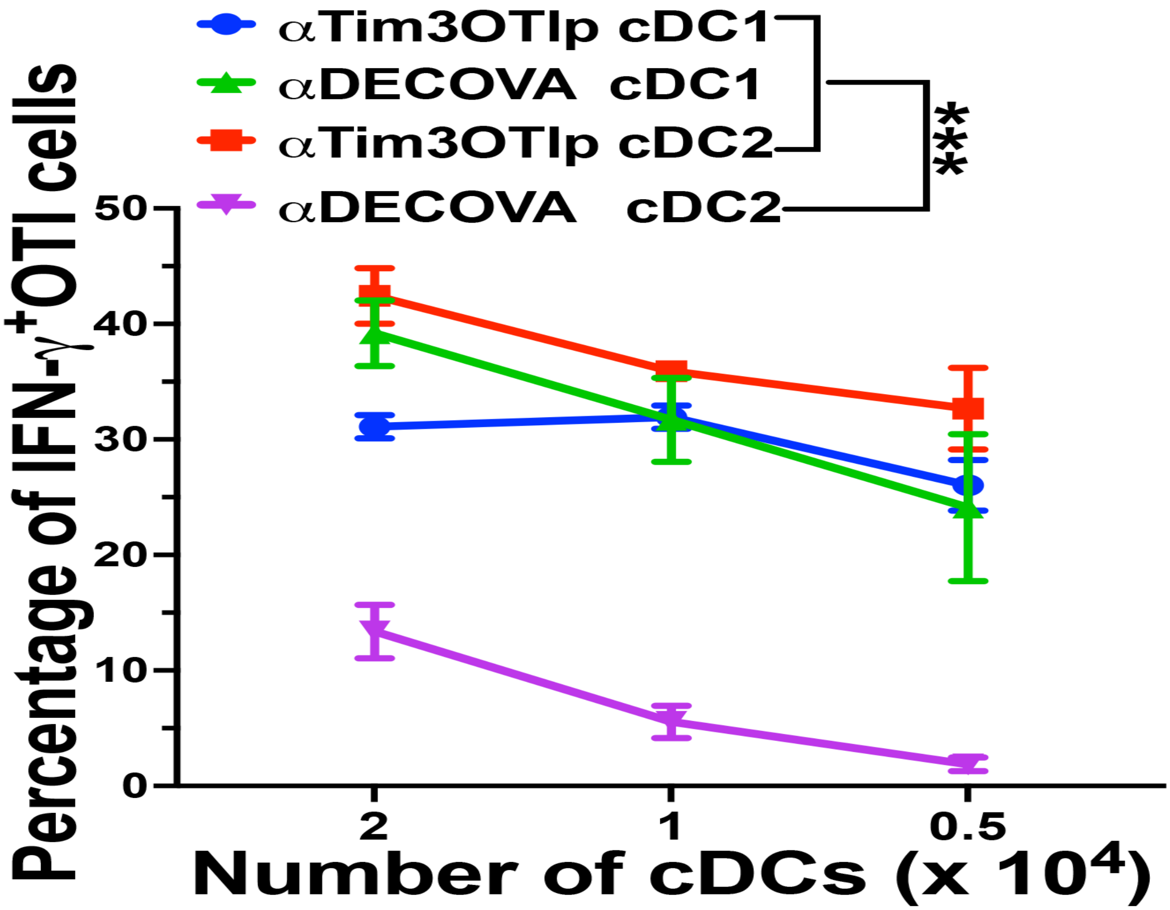

Figure S6. Anti-Tim-3OTIp but not anti-DEC-205OVA vaccines endow cDC2s with cross-priming capacity equivalent to or higher than cDC1s in vivo. WT mice were immunized with anti-Tim-3OTIp or anti-DEC-205OVA, and sorted cDC1s or cDC2s were cultured with naive OTI cells followed by examination of cross-priming on day 4 or 5. The percentages of IFN-γ^+ effectors out of total Thy1.1$^+$ OTI cells are shown. Two-way mixed ANOVA was performed to determine the difference between groups. $^{***}P < 0.001$. Data shown are representative of three experiments.

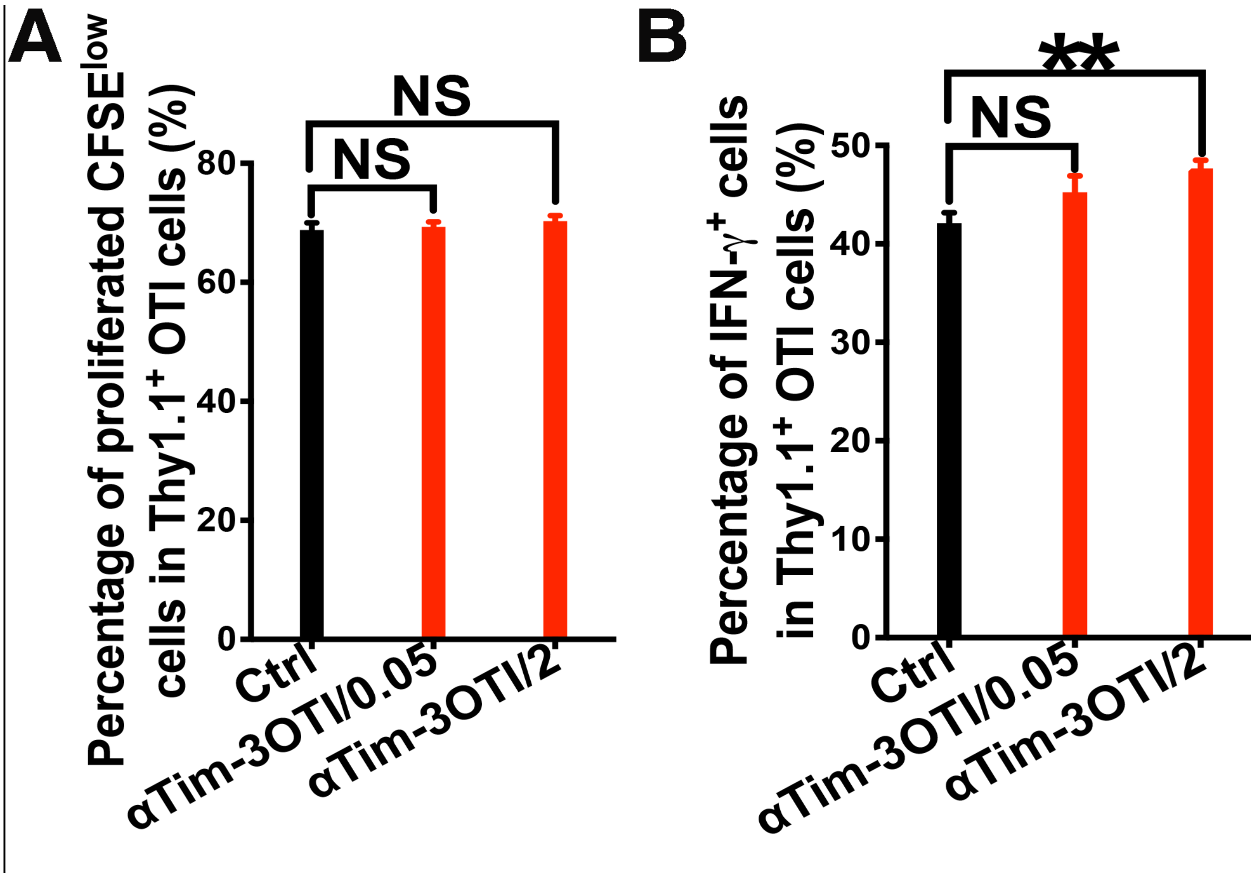

Figure S7. Anti-Tim-3OTIp enhances cross-priming primarily through DCs rather than direct effects on CD8 T cells. Splenic cDCs from WT mice were pulsed in vitro with anti–Tim-3OTIp (0.03 µg/mL) and then cocultured overnight with naïve Thy1.1$^+$ OTI CD8 T cells. Thy1.1$^+$ OTI cells were purified the following day, and cultured alone with graded concentrations of anti–Tim-3OTIp (0.05, and 2 µg/mL) as indicated. Cross-priming was examined at day 3 or day 4. The percentages of primed OTI cells that have proliferated **(A)** and the percentages of IFN-γ$^+$ effectors **(B)** out of total Thy1.1$^+$ OTI cells following 5 hour *in vitro* stimulation with $OTI_{257\text{-}263}$ with Brefeldin A (BFA) are shown. One-way ANOVA with Bonferroni-corrected post hoc t tests were used. $^{**}P < 0.01$, and NS > 0.05. Data shown are representative of three experiments.

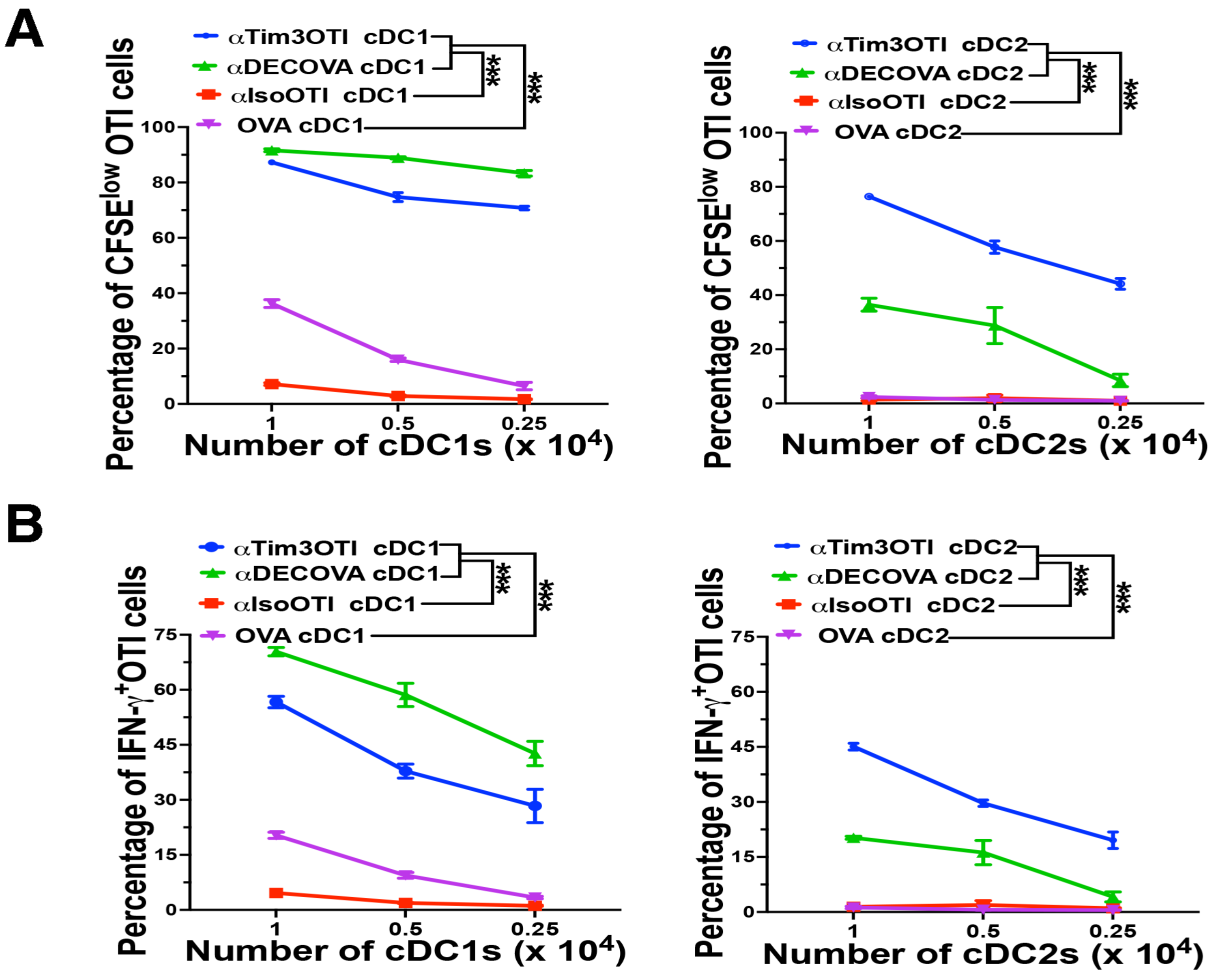

Figure S8. Anti-Tim-3OTIp uniquely endows cDC2s with capacity in cross-priming in vitro. Splenic cDCs from WT mice were pulsed in vitro with isotypeOTIp, anti-Tim-3OTIp, anti–DEC-205OVA (all at 0.03 μg/ml), or soluble OVA protein (1 μg/mL). Sorted cDC1s and cDC2 were cocultured with CFSE-labeled naïve OTI cells (5 x 10^4) at the indicated numbers of DCs, and cross-priming was examined at day 4 or 5. **(A)** The percentages of proliferated OTI cells out of total Thy1.1$^+$ OTI cells with cDC1s (left) or cDC2s (right), and **(B)** the percentages of IFN-γ$^+$ effectors out of total Thy1.1$^+$ OTI cells with cDC1s (left) or cDC2s (right) are shown. Two-way mixed ANOVA was performed to determine the difference between groups. $^{***}P <$ 0.001. Data shown are representative of three experiments.

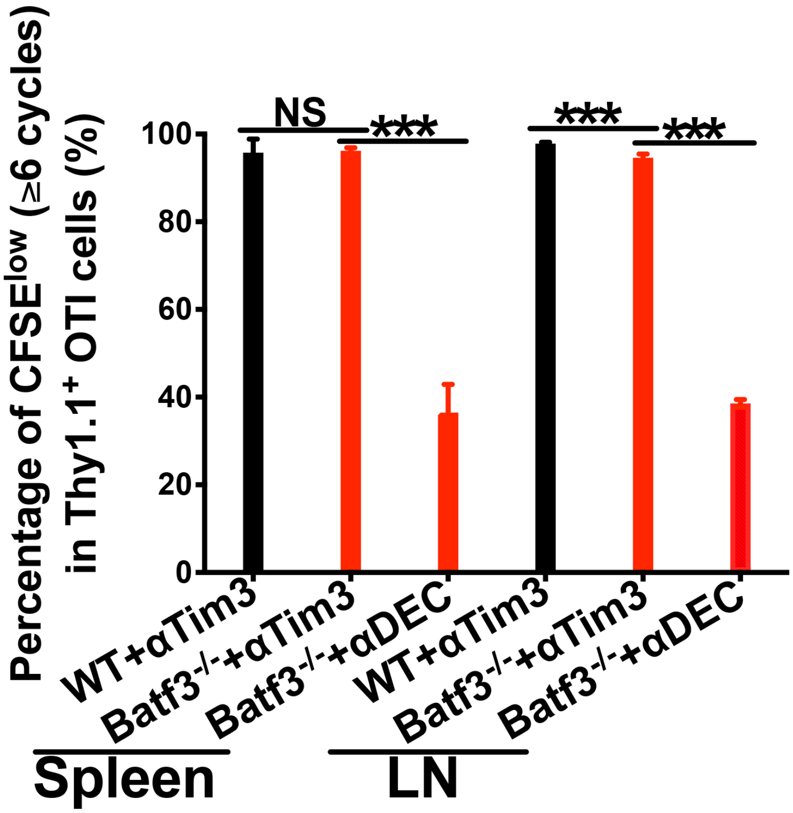

Figure S9. Anti-Tim-3OTIp vaccines induce cross-priming in the absence of cDC1s. Vaccination with anti-Tim-3OTIp induced cross-priming in Batf3$^{-/-}$ mice lacking cDC1s. WT and Batf3$^{-/-}$ mice (n=5) were immunized with anti-Tim-3OTIp, with Batf3$^{-/-}$ mice were also immunized with cDC1-targeted anti-DEC-205OVA. The percentages of OTI cells that have undergone proliferation (CFSE^{low}) out of total Thy1.1^{+} OTI cells are shown. Two-way mixed ANOVA was performed to determine the difference between groups in A, and one-way ANOVA with Bonferroni-corrected post hoc t tests were used for B. $^{***}P < 0.001$, and NS > 0.05. Data shown are representative of at least two experiments.

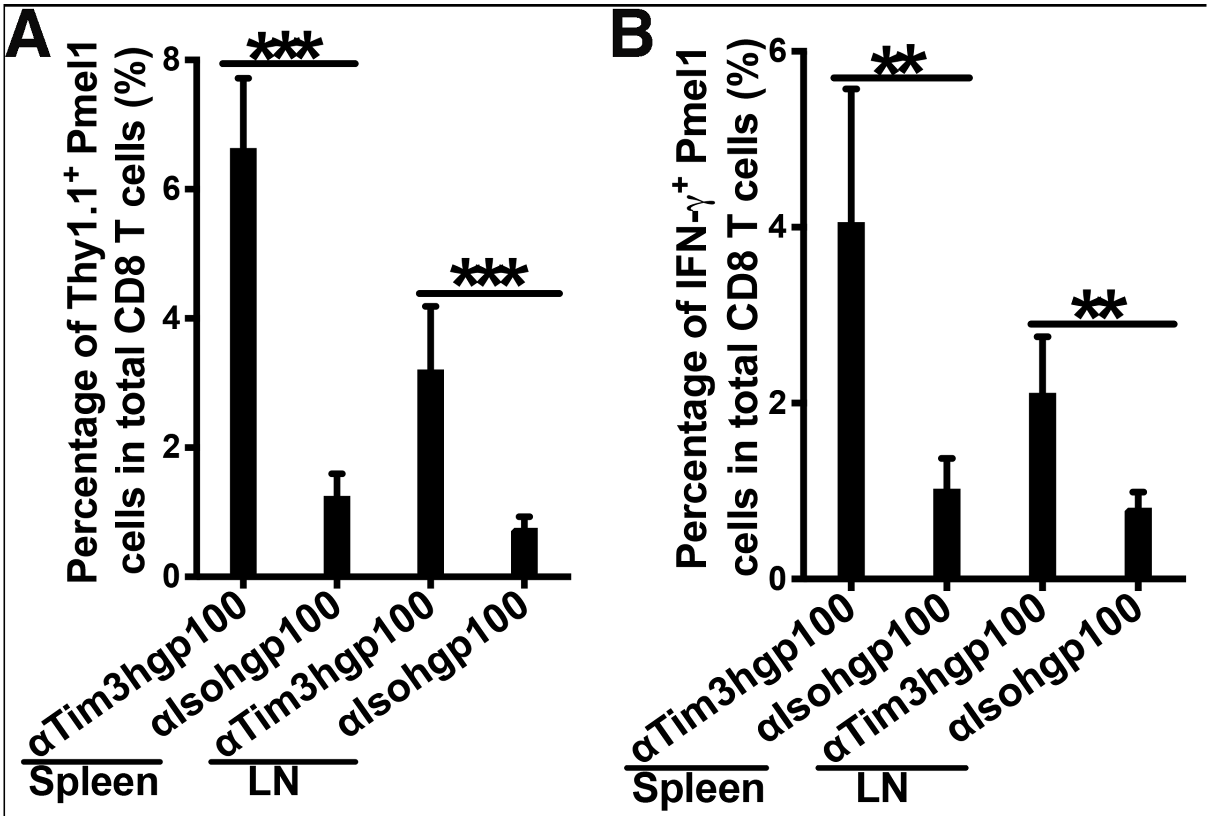

Figure S10. Tim-3-targeted vaccines with melanoma antigen human gp100 (anti-Tim-3hgp100) effectively induce cross-priming of gp100-specific CD8 T cells. WT mice (n=5) were immunized with either anti-Tim-3hgp100 or isotypehgp100 following adoptive transfer of naïve CFSE-labeled Thy1.1$^+$ Pmel1 cells, and cross-priming was examined at day 4 after immunization. The percentages of Thy1.1$^+$ Pmel1 cells in total CD8 T cells **(A)**, the percentages of IFN-γ$^+$ Pmel1 effectors out of total CD8 T cells **(B)** are shown. Student's t tests, $^{***}P < 0.001$, $^{**}P < 0.01$. Data are representative of four experiments.

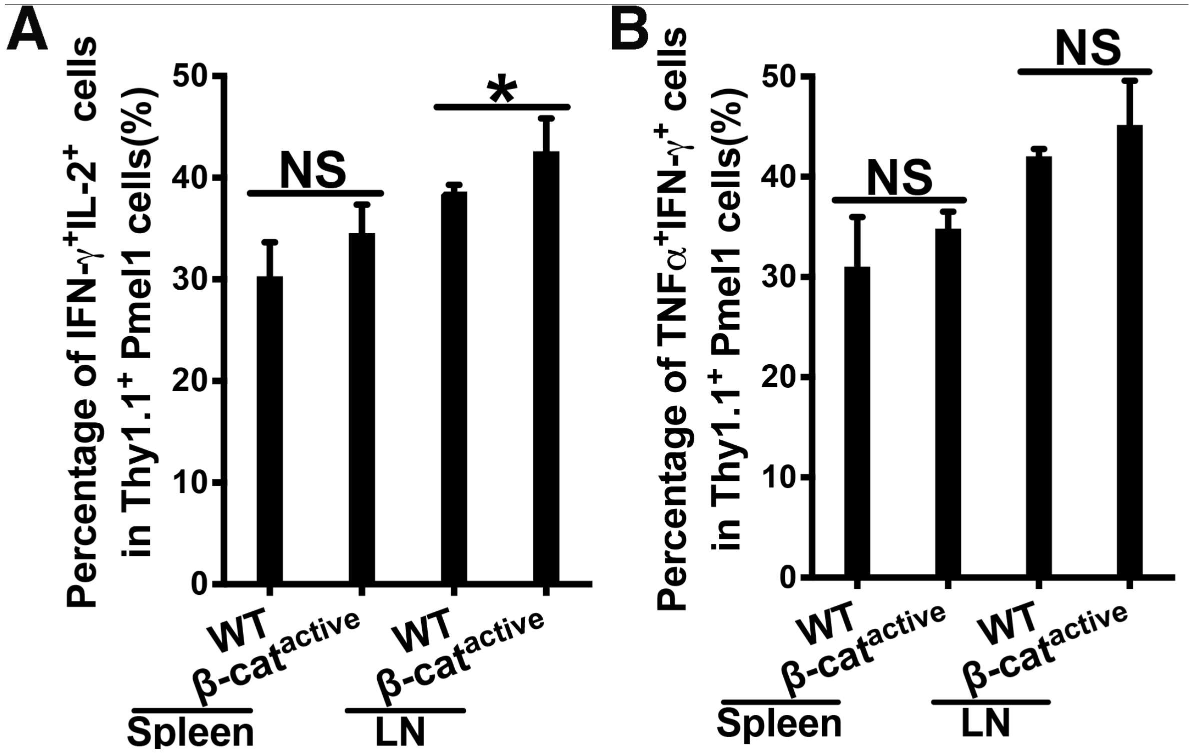

Figure S11. Tim-3-targeted vaccines with melanoma antigen human gp100 (anti-Tim-3hgp100) induce efficient cross-priming in CD11c-β-catenin^{active} mice. WT and CD11c-β-catenin^{active} mice (n=4-5) were immunized with anti-Tim-3hgp100, and cross-priming of adoptively transferred Thy1.1^{+} gp100-specific CD8 T (Pmel1) cells was examined at day 4 after immunization. The percentages of IFN-γ^{+}IL-2^{+} **(A)** and IFN-γ^{+}TNFα^{+} **(B)** effectors out of total Thy1.1^{+} Pmel-1 cells following 5 hour *in vitro* stimulation with hgp100$_{25\text{-}33}$ with Brefeldin A (BFA) are shown. Student's t tests, $^{*}P < 0.05$, and NS > 0.05. Data are representative of four experiments.

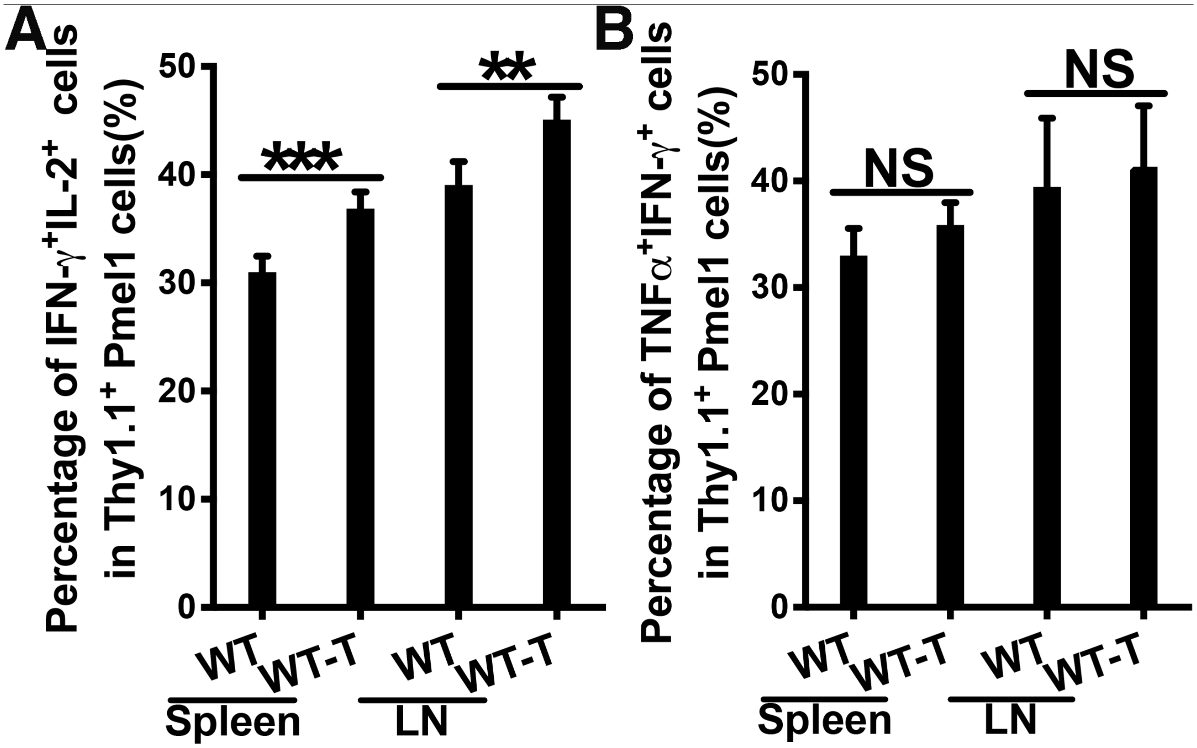

Figure S12. Vaccination with anti-Tim-3hgp100 induced comparable cross-priming in B16F10-bearing mice compared to tumor-naive WT mice. Tumor-naive and B16F10-bearing WT mice (n=5) were immunized with anti-Tim-3hgp100. The percentages of IFN-γ^+IL-2^+ **(A)** and IFN-γ^+TNFα^+ **(B)** effectors out of total Thy1.1^+ Pmel-1 cells following 5 hour *in vitro* stimulation with hgp$100_{25\text{-}33}$ with BFA are shown. Student's t tests, $^{***}P < 0.001$, $^{**}P < 0.01$, and NS > 0.05. Data are representative of two experiments.

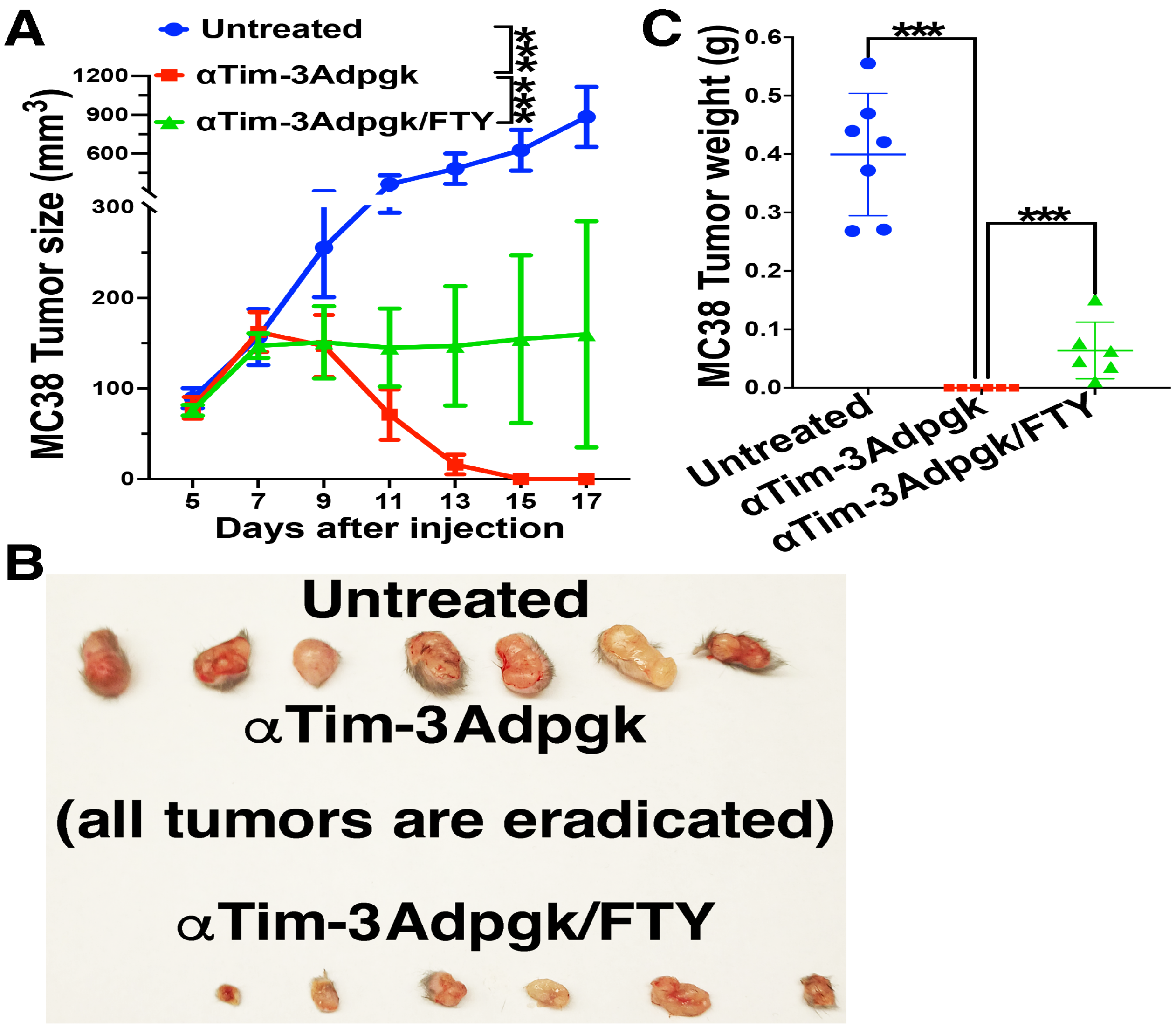

Figure S13. T cell trafficking from LN to tumors is required for anti-Tim-3Adpgk vaccination to eradicate existing tumors. MC38-bearing WT mice (n=6-7) were not treated, or immunized with anti-Tim-3Adpgk with or without FTY720 treatment when tumors are about 6-8mm (at day 5). Tumor sizes (A), photo of the tumors (B), and tumor weight (C) at the end of experiments are shown. Two-way mixed ANOVA was performed to determine the difference between groups in A, and one-way ANOVA with Bonferroni-corrected post hoc t tests were used for C. $^{***}P < 0.001$. Data are representative of two experiments.

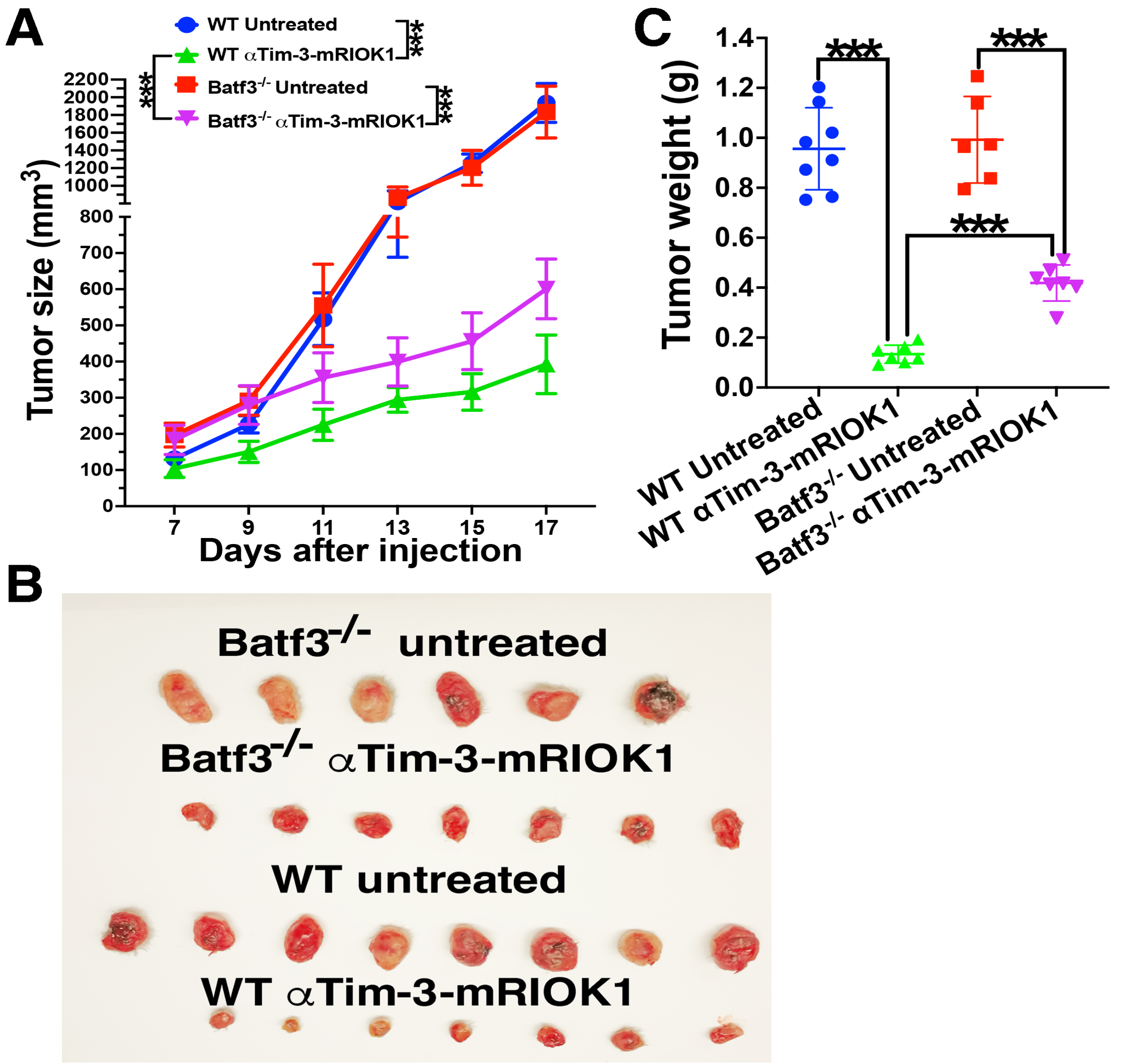

Figure S14. Tim-3-targeted neoantigen vaccines achieve anti-tumor efficacy in the absence of cDC1s in a Lewis Lung Carcinoma (LLC) model. LLC-bearing WT and Batf3$^{-/-}$ mice (n=6-8) were not treated, or immunized with anti-Tim-3mRiok1 (anti-Tim-3 conjugated with a long peptide containing mRiok1$_{312-319}$ epitope, a neoantigen for LLC) when tumor sizes were around 4-9 mm. Tumor sizes (Panel A), photo of the tumors (Panel B), and tumor weight (Panel C) at the end of experiments are shown. Two-way mixed ANOVA was performed to determine

the difference between groups for A, and one-way ANOVA with Bonferroni-corrected post hoc t tests were used for C. $^{***}P < 0.001$. Data are representative of two experiments.
